# A high quantum yield molecule-protein complex fluorophore for near-infrared II imaging

**DOI:** 10.1038/ncomms15269

**Published:** 2017-05-19

**Authors:** Alexander L. Antaris, Hao Chen, Shuo Diao, Zhuoran Ma, Zhe Zhang, Shoujun Zhu, Joy Wang, Alexander X. Lozano, Quli Fan, Leila Chew, Mark Zhu, Kai Cheng, Xuechuan Hong, Hongjie Dai, Zhen Cheng

**Affiliations:** 1 Department of Chemistry, Stanford University, Stanford, California 94305, USA; 2 State Key Laboratory of Virology, Key Laboratory of Combinatorial Biosynthesis and Drug Discovery (Wuhan University), Ministry of Education, Wuhan University School of Pharmaceutical Sciences, Wuhan 430071, China; 3 Molecular Imaging Program at Stanford (MIPS), Bio-X Program, and Department of Radiology, Canary Center at Stanford for Cancer Early Detection, Stanford University, Stanford, California 94305-5344, USA

## Abstract

Fluorescence imaging in the second near-infrared window (NIR-II) allows visualization of deep anatomical features with an unprecedented degree of clarity. NIR-II fluorophores draw from a broad spectrum of materials spanning semiconducting nanomaterials to organic molecular dyes, yet unfortunately all water-soluble organic molecules with >1,000 nm emission suffer from low quantum yields that have limited temporal resolution and penetration depth. Here, we report tailoring the supramolecular assemblies of protein complexes with a sulfonated NIR-II organic dye (CH-4T) to produce a brilliant 110-fold increase in fluorescence, resulting in the highest quantum yield molecular fluorophore thus far. The bright molecular complex allowed for the fastest video-rate imaging in the second NIR window with ∼50-fold reduced exposure times at a fast 50 frames-per-second (FPS) capable of resolving mouse cardiac cycles. In addition, we demonstrate that the NIR-II molecular complexes are superior to clinically approved ICG for lymph node imaging deep within the mouse body.

## Abstract (web summary)

Near-infrared (NIR) fluorescence imaging >1,000 nm allows deep tissue imaging, but available organic dyes display poor brightness and temporal resolution. Here, the authors synthesize a NIR dye that, upon binding serum proteins, exhibits a 110-fold increase in intensity, giving an 11% quantum yield.

Within the last 5 years, the development of new fluorophores emitting in the second near-infrared widow (NIR-II) at wavelengths ranging from 1,000 to 1,700 nm has allowed visualization of deep anatomical features with an unprecedented degree of clarity^1^,^2^,^3^,^4^,^5^,^6^,^7^,^8^,^9^,^10^,^11^. Light scattering and attenuation as well as background autofluorescence all decrease when imaging at progressively longer wavelengths, which have enabled non-invasive through-skull imaging of brain vasculature, detecting tumours at depths of ∼4 mm in the brain, resolving blood flow dynamics resulting from cardiac waveforms, and assessing blood flow anomalies in cardiovascular disease and traumatic brain injury^1^,^2^,^3^,^10^. However, the majority of NIR-II fluorophores suffer from low quantum yields as the generation of long-wavelength photons require low bandgap materials in which non-radiative decay processes tend to dominate over radiative photon emission^12^. In the visible or NIR-I (∼750–1,000 nm) regions quantum yields of ∼80% and ∼10%, respectively, are common yet those in the NIR-II are typically in the range of ∼0.01–1.4% with the exception of lead sulfide quantum dots^1^,^13^,^14^,^15^,^16^. The dearth of high quantum yield NIR-II fluorophores has hindered fully reaching the potential of deep-imaging penetration depths in this window and caused poor temporal resolution as long exposure times are needed to compensate for low brightness. A clinically translatable high quantum yield organic NIR-II fluorophore would open up many exciting possibilities for biomedical fluorescence imaging.

While most current NIR-II fluorophores consist of inorganic semiconducting nanomaterials, the development of long-wavelength organic dyes similar in functionality and biocompatibility to those in the visible and NIR-I should allow NIR-II imaging to transition rapidly into a clinical setting and the broader research community. Donor–acceptor–donor (D–A–D) dyes are a promising class of small-molecule fluorophores for bioimaging whose emission extends past 1,000 nm^11^,^17^. Traditionally used for semiconducting organic electronics such as OLEDs and dye sensitized solar cells, this highly tunable dye architecture is built around a strong electron acceptor such as benzo[1,2-c:4,5-c′]bis([1,2,5]thiadiazole) (BBTD) to which strong electron donors are wired through *π*-spacers to lower the energy gap. Molecular engineering of the energy difference between the highest occupied molecular orbital (HOMO) and lowest unoccupied molecular orbital (LUMO) levels allows wavelength tuning of emission peaks spanning the NIR-II. However, the hydrophobic nature of these dyes requires additional functionalization to increase aqueous solubility. We recently reported the first organic dye for NIR-II imaging, a carboxylated D–A–D dye termed CH1055, which required PEGylation if not conjugated to a hydrophilic protein^2^. Although CH1055-PEG demonstrated excellent pharmacokinetics, a low quantum yield of ∼0.3%, while typical of NIR-II fluorophores, leaves ample room for further improvement.

Herein, we demonstrate that changing the functional groups from carboxylic to sulfonic acid results in a completely water-soluble organic NIR-II dye (CH-4T) that readily forms supramolecular assemblies with plasma proteins to produce a brilliant increase in fluorescent brightness^18^. The fluorescence intensity of CH-4T increases ∼50-fold in serum compared to phosphate-buffered solution (PBS) and revealed an impressively high quantum yield of up to ∼5%. Further, simply heating the dye to 70 °C for 10 min in serum boosted its quantum yield up to 11%, affording the highest reported quantum yield to date for a clinically suitable NIR-II fluorophore. Optimizing the brightness of CH-4T through protein complexation prior to injection allowed for the fastest video-rate imaging in the NIR-II with ∼1.5–2 ms exposure times and ultra-fast 50 frames-per-second (FPS) dynamic imaging capable of resolving cardiac cycles with unprecedented temporal resolution. In addition, we demonstrate that lymph nodes deep within the mouse (∼5–8 mm) are clearly imaged in the NIR-II using the CH-4T complex with imaging performance superior to indocyanine green (ICG).

## Results

### Synthesis and optical characterization

**Figure 1:**
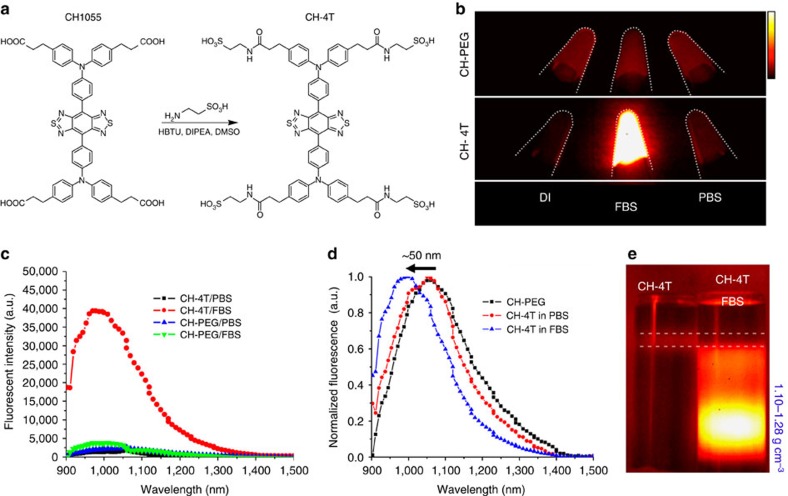
Synthesis and optical characterization of CH-4T. (**a**) Chemical structure of CH1055 and the one-step sulfonation to produce CH-4T. (**b**) NIR-II fluorescent images (10 ms, 1100LP) of CH-PEG and CH-4T in deionized water, FBS and PBS at equivalent absorbance of OD 0.1 at 808 nm. Colourbar next to **b** ranges from 1,000 to 15,000. (**c**) Fluorescent emission spectra of CH-PEG and CH-4T in FBS, PBS after excitation at 808 nm. (**d**) Normalized fluorescent emission spectrum of CH-PEG in PBS and CH-4T in FBS, PBS demonstrating ∼50 nm hypsochromic shift. (**e**) NIR-II fluorescent image (1000LP, 10 ms) of centrifuge tubes after sucrose DGU with free CH-4T dye on the left and CH-4T pre-mixed with FBS on the right. Dotted white line indicates buoyant density region of unbound CH-4T as seen in free dye band in left tube. Bright regions in right centrifuge tube correspond to CH-4T–protein complexes that are found at the higher buoyant densities typical of proteins (∼1.10–1.28 g cm^−3^).

**Figure 2:**
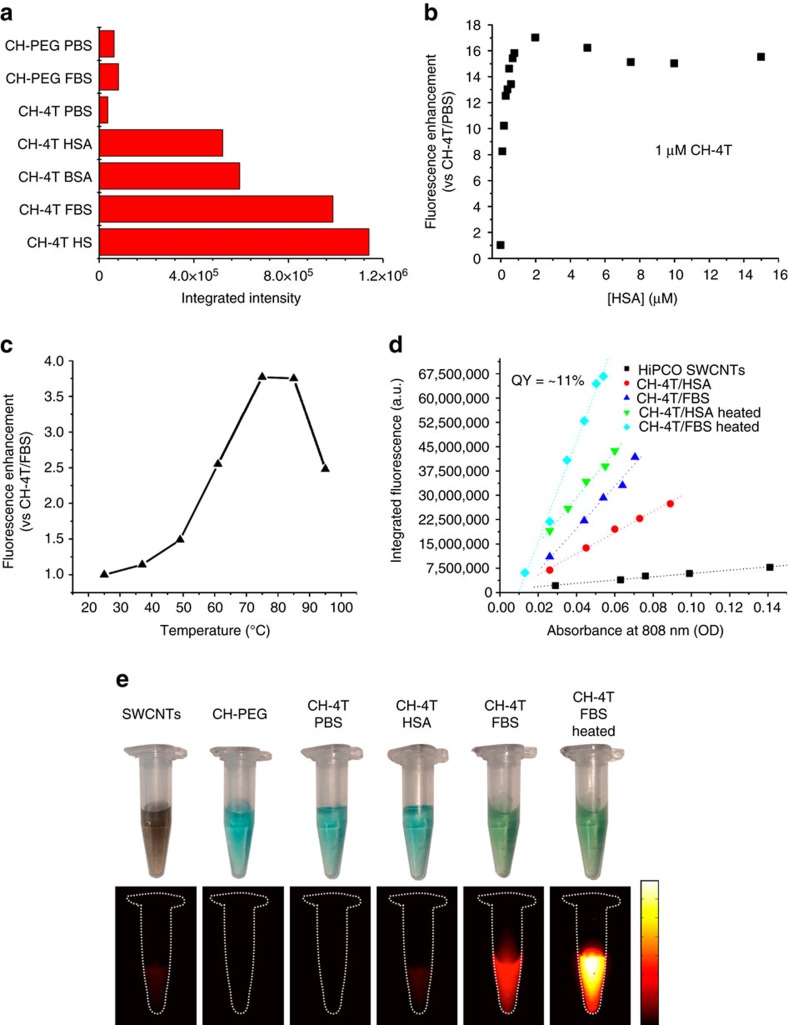
Ultra-high quantum yield CH-4T–protein complexes. (**a**) Fluorescent brightness of CH-PEG, CH-4T in protein solutions at 5:1 protein-to-dye molar ratio (**b**) Fluorescent titration for constant 1 μM of CH-4T in increasing HSA receptor concentration. (**c**) Plot demonstrating fluorescence brightening through heating for 10 min of CH-4T pre-mixed with FBS and horn sonicated FBS. (**d**) Plot of the integrated fluorescence spectrum of CH-4T pre-mixed with HSA, FBS before and after heating at 70 °C for 10 min at five different concentrations. Linear fits were used to calculate quantum yield by comparing the slopes to reference HiPCO SWCNTs (QY=0.4%) (**e**) Photographs and corresponding NIR-II fluorescent images (10 ms, 1000LP) of carbon nanotubes, CH-PEG and CH-4T in HSA, FBS and heated FBS. Photographs were taken at an absorbance value of OD 1 at 808 nm, while NIR-II fluorescent images were at the lower concentration of OD 0.1. Colourbar next to **e** ranges from 4,000 to 40,000.

Synthesis of the sulfonated small-molecule organic dye (CH-4T, 1.4 kDa, Fig. 1a) was achieved through amide bond formation between the carboxylated CH1055 and taurine by *O*-(benzotriazol-1-yl)-*N,N,N′,N′*-tetramethyluronium hexafluorophosphate (HBTU), *N*,*N*-diisopropylethlamine (DIPEA) coupling in DMSO (see Supplementary Methods for complete synthesis details). Taurine is a common biomolecule found throughout the body containing both an amine and sulfonate group, thus allowing facile modification of CH1055. After conjugation, CH-4T was purified with HPLC and the eluted fractions analysed by matrix-assisted laser desorption ionizing time-of-flight mass spectrometry (MALDI-TOF-MS) to collect CH1055 conjugated to four taurine moieties (Supplementary Fig. 1). An nuclear magnetic resonance (NMR) spectrum further confirmed the synthesis and purity of CH-4T (Supplementary Fig. 2). An ultraviolet-visible absorbance spectrum revealed a strong peak at ∼738 nm (Supplementary Fig. 3). The relative fluorescent brightness of CH-4T was investigated by matching the absorbance at 808 nm (optical density (OD) 0.1) in deionized water (DI), PBS and fetal bovine serum (FBS) and imaging the vials on an indium-gallium-arsenide (InGaAs) camera under 808 nm excitation. The fluorescent brightness of CH-4T in DI water was marginally higher than in PBS, yet a drastic 35-fold increase in fluorescence in the range of 900–1,500 nm was observed after mixing free CH-4T dye with FBS (10 ms, 1100LP; Figs 1b and 2e for higher image dynamic range; see Methods for details on CH-4T spectroscopy). In contrast, CH1055-PEG mixed at equivalent concentrations in identical biological media produced a uniform NIR-II brightness across all solutions (Fig. 1b). A fluorescent emission spectrum collected for CH-4T and CH-PEG in both PBS and FBS under an excitation of 808 nm (using a NIR-II spectrophotometer that compensated for the variability in the detector quantum efficiency at different wavelengths (Fig. 1c)) demonstrated a higher fluorescence brightness of CH-4T in serum than CH-PEG by 16-fold, again suggesting the CH-4T dye became brilliantly bright in the presence of serum proteins. CH-4T mixed with FBS from various sources all produced virtually the same level of fluorescence enhancement regardless of manufacturer (Supplementary Fig. 4).

To investigate CH-4T binding to biomacromolecules, the normalized fluorescence emission spectrum of CH-PEG in PBS was compared to CH-4T in both PBS and FBS. The fluorescence emission peak of CH-PEG and CH-4T in PBS occurred at ∼1,055 nm with emission from 900 to ∼1,400 nm as expected from previous CH1055 spectroscopic measurements. However, CH-4T in serum yielded a hypsochromic shift of ∼50 nm which is indicative of non-covalent binding between the dye and proteins through hydrophobic van der Waals's interactions as well as ion pairing between the sulfonate groups and cationic amino acid residues such as histidine, lysine and arginine^19^.

To further verify the formation of CH-4T complexes with plasma proteins, the free dye and CH-4T pre-mixed with FBS (CH-4T/FBS) were subjected to isopycnic density gradient ultracentrifugation (DGU) (Fig. 1e; see Methods for complete DGU details). A linearly changing sucrose density gradient (1.10–1.28 g cm^−3^) was established along the length of the centrifuge tube that corresponds to the average buoyant density range of proteins. During ultracentrifugation, macromolecules migrate to a position in the gradient where their buoyant density matches that of the surrounding gradient. Free CH-4T dyes formed a distinct band near ∼1.10 g cm^−3^, while for CH-4T/FBS a heterogeneous distribution of protein-bound fluorescence-enhanced CH-4T was observed throughout the density gradient with a bright region at the bottom of the tube (∼1.20 g cm^−3^). The absence of fluorescence in the buoyant density range corresponding to free CH-4T in the centrifuge tube containing CH-4T/FBS indicates near-zero free dye and a strong protein binding affinity^20^.

### Optimizing the brightness of CH-4T–protein complexes

To develop an ultra-bright NIR-II probe by exploiting CH-4T–protein interactions, common serum proteins were added in excess to CH-4T to observe the increase in fluorescence enhancement under the NIR-II camera. Both human and bovine serum albumin (HSA and BSA) demonstrated strong fluorescence enhancement by 17-fold yet the intensity was ∼2 × lower than the dye in serum (Fig. 2a). Even weaker CH-4T fluorescence occurred when mixed with IgG antibodies. A fluorescence titration was performed by increasing the HSA concentration while keeping CH-4T at a constant 1 μM to determine the approximate binding stoichiometry. As seen in Fig. 2b, the maximum fluorescence intensity occurred at approximately a 2:1 HSA:CH-4T molar ratio.

As the brightness of CH-4T seems highly dependent on optimal binding between the dye and protein surfaces, heating of the complexes was attempted with the hopes of exposing CH-4T to typically inaccessible hydrophobic domains located in the protein interior. After heating for 10 min, the brightness of the vials containing the CH-4T and FBS mix increased steadily with temperature until ∼75–85 °C. However, heating to temperatures above ∼85 °C caused a precipitous drop in fluorescent intensity. Significant aggregation was observed for temperatures above ∼90 °C, while a minimal amount of aggregation at lower temperatures was removed through centrifugation post-heating (30 min, 15,000 r.p.m.). Heating virtually every dye–protein complex caused a marked increase in fluorescent intensity with a similar ∼50 nm hypsochromic shift from ∼1,050 to ∼1,000 nm. After returning to room temperature, the thermally stabilized dye–protein complexes maintained their boosted brightness when compared to solely mixed solutions.

The CH-4T–protein complexes represented the brightest fluorescent agent based on organic molecules with an emission peak in the ∼1,000–1,100 region of the NIR-II. Comparing the brightness of fluorophores in vials and cuvettes with the NIR-II camera may cause errors due to inter-filter effects, while the hypsochromic emission peak shift coupled with the nonlinear camera quantum efficiency as a function of wavelength necessitates a more rigorous measurement. The relative fluorescence brightness of CH-4T/FBS heated to 70 °C for 10 min, termed CH-4T/FBS-HT, was 28-fold, 36-fold and 22-fold brighter than carbon nanotubes (QY=0.4%; IR-26=0.5%), CH-PEG (QY=0.3%; IR-26=0.5%) and IR-26 (QY=0.5%) in DCE, respectively, with a 110-fold brightness difference between CH-4T/PBS and CH-4T/FBS-HT when measured on a wavelength-corrected NIR-II spectrophotometer (see Methods; Supplementary Fig. 5; Supplementary Tables 1 and 2 for enhancement calculations). Due to recent discrepancies in the reported quantum yield of the IR-26 reference fluorophore (QY_IR26_∼0.05–0.5%; see Discussion), the absolute quantum yields of CH-4T/PBS, CH-4T/FBS and CH-4T/FBS-HT are in the range of 0.0098–0.098%, 0.48–4.8% and 1.8–10.8%, respectively. The NIR-II fluorescent images (1000LP, 10 ms) in Fig. 2e qualitatively shows the disparate brightness levels between the brilliantly fluorescent CH-4T–protein complexes and current NIR-II contrast agents such as single-walled carbon nanotubes (SWCNTs) and CH-PEG, all with matching absorbance at 808 nm (OD 0.1).

### Ultra-fast *in vivo* imaging with CH-4T

**Figure 3:**
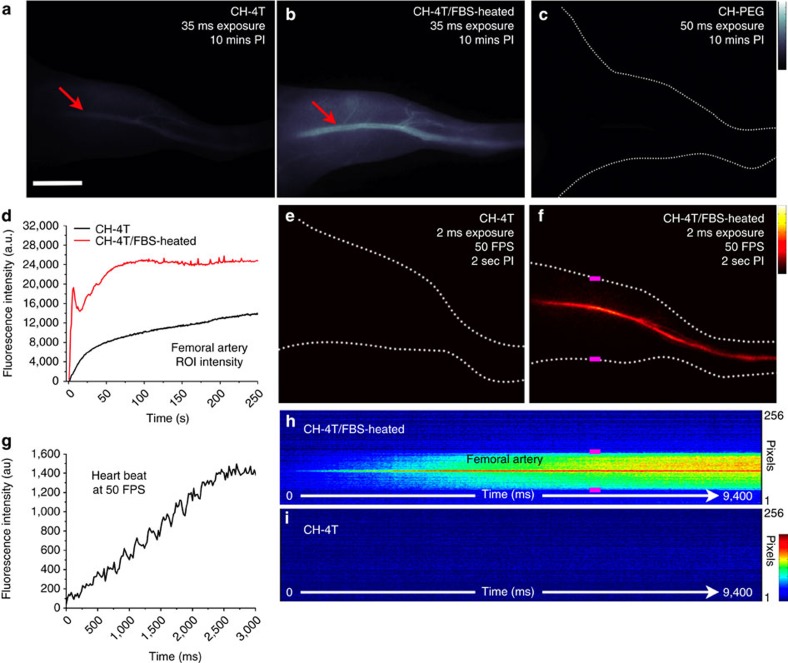
Free CH-4T versus CH-4T–protein complexes for NIR-II hindlimb vasculature imaging. NIR-II fluorescent images of mouse hindlimb vasculature 10 min post-injection of (**a**) free CH-4T, (**b**) CH-4T heated at 70 °C for 10 min in FBS (CH-4T/FBS-HT) and (**c**) CH-PEG. All injection doses were normalized to an absorbance of OD 3.2 at 808 nm with an exposure time of 35 ms, 50 ms for CH-4T, CH-PEG, respectively. Scale bar in **a** of 0.5 cm and corresponds to **a**–**c**,**e**,**f**. Colourbar next to **c** ranges from 0.5 to 5 × 10^4^ and corresponds to **a**–**d**. Fluorescent intensity of integrated ROI region on the hindlimb femoral artery plotted for 250 s post-injection during video-rate imaging of free CH-4T and CH-4T/FBS-HT (35 ms exposure time). NIR-II fluorescent images 2 s post-injection of **e** free CH-4T and **f** CH-4T/FBS-HT during ultra-fast 50 FPS imaging. Colourbar next to **f** ranges from 500 to –5,000 and corresponds to **e**,**f**,**g**. Fluorescent intensity of integrated ROI region on the femoral artery for 3 s post-injection demonstrating a clearly resolvable cardiac cycle. The fluorescence intensity of a single vertical row of pixels during 50 FPS imaging in **e**,**f** was plotted against time for **h** CH-4T/FBS-HT and **i** free CH-4T during 9.4 s post-injection. Pink bars in **f**,**h** mark the edges of the hindlimb. The femoral artery is clearly visible as the solid red streak in **h** only after injection of pre-bound CH-4T in heated FBS. Colourbar next to **i** ranges from 0 to 2,000 and corresponds to **h**,**i**. All NIR-II images and fluorescent intensity profiles for **e**–**i** were collected at a 2 ms exposure time. All NIR-II imaging was performed with a 1,000 nm long-pass emission filter.

To investigate the *in vivo* optical properties of free CH-4T dyes and the brightness optimized CH-4T–protein complexes, video-rate imaging (1000LP, 35 ms exposure time) of mouse hindlimb vasculature was performed after an intravenous injection of free CH-4T and CH-4T/FBS-HT at equivalent dosages (90 μg, 100 μl at OD 3.2 at 808 nm) under 808 nm excitation at a power density of 140 mW cm^−2^. After 10 min post-injection, a clear difference in the fluorescent intensity of the femoral vasculature was discerned as seen in Fig. 3a,b indicating that the higher quantum yield of pre-made complexes of CH-4T/FBS-HT compared to CH-4T that complexed with serum proteins *in vivo* in circulating blood upon injection of free CH-4T generated a brighter contrast agent. The ∼2 × brighter femoral artery of CH-4T/FBS-HT injected mice compared to those injected with free dye correlates with the ∼2–3 × boosted solution brightness difference observed between CH-4T/FBS-HT and the free dye mixed in serum. For comparison, much weaker fluorescence was observed at any time from a mouse injected with an equivalent CH-PEG dosage (100 μl at OD 3.2 at 808 nm; Fig. 3c), even at higher exposure times of 50 ms, since CH-PEG showed no complexing with serum proteins to afford enhanced fluorescence *in vivo*.

While the CH-4T/FBS-HT complex yielded a ∼2 × increase in the steady-state vessel fluorescent intensity compared to free dye, the benefits of pre-optimizing the brightness of the CH-4T–protein complexes are highly desired for imaging immediately post-injection. In Fig. 3d, the integrated intensity of a background subtracted region of interest (ROI) region in the femoral artery was plotted for the first 250 s post-injection for CH-4T and CH-4T/FBS-HT (position of ROI region indicated by red arrows in Fig. 3a,b). During the first 10 s post-injection, a strikingly stronger rise in fluorescence was observed from the femoral artery of mice injected with the pre-brightened complex compared to free CH-4T. In contrast, the gradual increase in fluorescent intensity observed from the femoral artery of the mouse injected with free CH-4T occurred as the brightening kinetics of the free dye required longer periods of time to binds to serum proteins during circulation to reach its full fluorescent potential brightness. By dividing the fluorescent intensity of the femoral artery of the pre-bound versus free dye (Supplementary Fig. 6), earlier time points demonstrate a ∼33-fold higher brightness that equilibrates to the steady-state brightness difference of ∼2-fold at ∼200 s. After this point, the vessel intensities of mice injected with free CH-4T have formed stable complexes and reached their steady-state maximum fluorescent brightness. The brightness difference between the femoral artery of CH-4T and the pre-brightened complex injected mice now matches the brightness difference seen at longer time points post-injection in Fig. 3a,b. In strong contrast to CH-4T, CH-PEG at an equivalent dose produced a barely detectable increase in fluorescent signal in the femoral artery after injection that remained at a constant low fluorescent intensity (Supplementary Fig. 7).

The markedly higher brightness of pre-bound CH-4T circulating in the vascular system during the first seconds post-injection can be exploited for high temporal resolution imaging of hemodynamic processes. A stark difference between injecting free CH-4T and CH-4T/FBS-HT (250 μg CH-4T/mouse) is clearly seen in the selected frames at equivalent time points during ultra-fast 50 FPS video imaging using exposure times of only 1.5–2 s (Fig. 3e,f) with an instrument overhead time of ∼19 ms. At 2 s post-injection, no fluorescence is noted from the vasculature of mice injected with free CH-4T due to the slow brightening kinetics of the dye *in vivo*. In contrast, the femoral artery is clearly resolved during 50 FPS imaging when injecting CH-4T/FBS-HT (Fig. 3e). Plotting the integrated ROI intensity of the femoral artery for 3 s post-injection of CH-4T/FBS-HT allows for precise imaging of a cardiac cycle where intensity peaks corresponding to ventricular ejections (Fig. 3g) are resolved with much higher resolution compared to previous attempts with lower quantum yield semiconducting NIR-II polymers (see Supplementary Fig. 8 for comparison)^1^. The fluorescent intensity from a single row of pixels crossing the hindlimb was selected from each frame for the first 9.4 s of the 50 FPS videos that generated the images seen in Fig. 3e,f and plotted as a function of time (Fig. 3h,i). The pink position markers denote the edges of the hindlimb in Fig. 3f,h. Plotting the fluorescent intensity line profile of a single row of pixels over time clearly shows that the blood flow front along with the femoral artery, visualized as the strong red streak in Fig. 3h, can easily be discerned with the CH-4T/FBS-HT contrast agent yet virtually no fluorescence can be observed during the first ∼10 s when injecting unbound CH-4T in Fig. 3i.

### Deep lymph node imaging with CH-4T complexes

**Figure 4:**
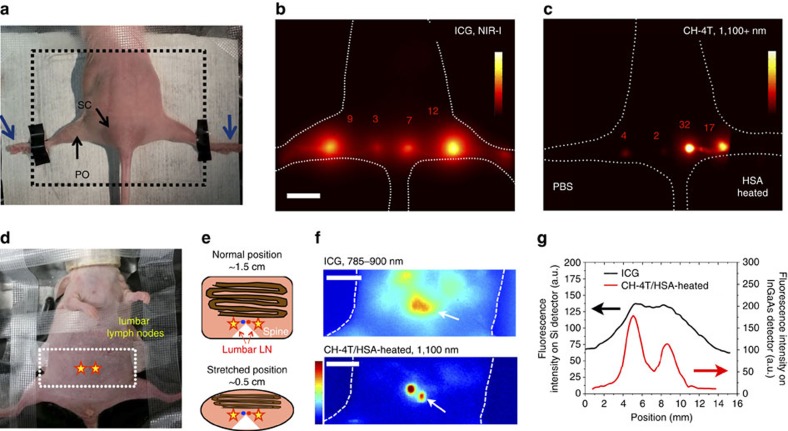
Lymph node imaging with ultra-bright CH-4T–protein complexes. (**a**) Photograph depicting nude mouse in prone position for imaging popliteal and sacral lymph nodes in **b**,**c**. Fluorophore injection sites indicated by blue arrows next to footpad. (**b**) NIR-I fluorescent image ∼30 min post-injection of 50 μM ICG (50 ms exposure; colourbar ranges from 4,000 to 40,000). (**c**) NIR-II fluorescent image ∼30 min post-injection of CH-4T/PBS injected in left foot and CH-4T/HSA-HT in right foot at equivalent dosages of 50 μM CH-4T (100 ms exposure; colourbar ranges from 4,000 to 55,000). 1 cm scale bar in **b** corresponds to both **b**,**c**. (**d**) Photograph depicting nude mouse in a stretched position to narrow the distance between lumbar lymph nodes and mouse surface. Stars indicate position of lumbar lymph nodes and white box corresponds to imaging field of view (**e**). Illustration of mouse anatomy surrounding lumbar lymph nodes demonstrating a change in imaging depth from ∼1.5 to ∼0.5 cm. White triangle corresponds to the spine with red and blue circles representing descending aorta and inferior vena cava, respectively. Brown lines represent intestines. (**f**) NIR-I/II fluorescent images of lumbar lymph nodes after 50 μM of ICG and CH-4T/HSA-HT were injected sequentially into both footpads at an exposure time of 300 and 400 ms, respectively. ICG and CH-4T imaging performed with a silicon and InGaAs camera, respectively, with identical 785 nm excitation laser conditions. Colourbar spans 0–1,500 for ICG and 0–8,000 for CH-4T/HSA-HT. Scale bars, 1 cm. (**g**). White arrows correspond to direction of the **g** fluorescent cross-sectional intensity profile of lumbar lymph nodes on each detector.

To further investigate the advantages of high-brightness contrast agents for biomedical fluorescence imaging, the imaging quality of lymphatic vasculature and nodes were compared in both the first and second NIR windows. The popliteal and sacral lymph nodes were imaged in nude mice in a prone position with fluorophores injected in both footpads as seen in Fig. 4a. ICG dispersed in DI water at 50 μM was utilized as a NIR-I contrast agent and lymph node-to-background ratios of 8.6±2.9 were observed (Fig. 4b) which is consistent with reported values^21^. Although ICG can form complexes when mixed with proteins such as HSA, the benefits of the premixed ICG/HSA complex compared to free ICG in terms of lymph node detection efficiency is unclear^21^,^22^. Lymph node fluorescence from ICG and ICG/HSA-HT likewise produced nearly identical intensities as seen in Supplementary Fig. 9. However, a very notable difference in brightness of ∼8-fold is observed between free CH-4T injected on the left footpad and CH-4T/HSA-HT injected on the right side footpad (Fig. 4c) at equivalent 50 μM dosages immediately post-injection. The disparate fluorescent intensities of the popliteal and sacral lymph nodes for free CH-4T and CH-4T/HSA-HT demonstrated the need for premixing to form ultra-bright complexes for NIR-II lymphography. CH-4T/HSA-HT resulted in lymph node-to-background ratios of 29±7.6 with significantly sharper lymph node features with a popliteal/sacral FWHM of 2.52±0.23 mm/1.61±0.11 mm compared to 4.86±0.17 mm/4.4±0.29 mm with ICG in the NIR-I.

To demonstrate the enhanced clarity of features deep inside the body by imaging in the NIR-II, the lumbar lymph nodes were visualized after the belly was stretched to reduce the distance between the lumbar lymph nodes and the surface of the mouse (Fig. 4d). The lumbar lymph nodes sit on either side of the spine, laterally of the descending aorta bifurcation when observing the mouse in a supine position and are connected to a lymphatic channel responsible for draining the footpad and leg (see Supplementary Fig. 10 for lymphatic system details). Without contortion, the distance between the lumbar lymph nodes and the surface approaches ∼1–1.5 cm, the bulk of which is mostly comprised of the intestines as shown in the illustration in Fig. 4e. Stretching the belly provides a fixed imaging depth of ∼0.5–0.8 cm and allows sufficient lymph node fluorescent signal in both windows (see Methods for details).

ICG (50 μM) was injected into the footpad and digits on both sides; afterwards the mouse was fixed physically and stretched on the imaging platform until the lumbar lymph nodes were visualized with a silicon detector in the NIR-I (300 ms, 785–900 nm; for imaging set-up details see Supplementary Figs 11 and 12). The subsequent injection of CH-4T/HSA-HT (50 μM) on the same mouse in an identical manner and imaging on the InGaAs detector (400 ms, 1,100+ nm) demonstrated a striking increase in the resolution of the lumbar lymph nodes as seen in Fig. 4f. Imaging with the CH-4T–protein contrast agent allowed the resolution of individual nodes with a signal-to-background (SBR) ratio of ∼13 as opposed to the diffuse conjoined features observed in the NIR-I with an SBR of ∼2.5. The enhanced clarity is reflected in the fluorescence cross-sectional intensity profile of the lumbar lymph nodes in both windows in Fig. 4g as well as the ∼5 × lower background autofluorescence levels in the NIR-II. Imaging in both windows was performed with a 785 nm laser (16 mW cm^−2^) to provide a constant excitation power density for both dyes.

## Discussion

Imaging in the second near-infrared window allows optical imaging to peer deeper into the body with more clarity than any other wavelength regime^22^,^23^,^24^,^25^,^26^,^27^. However, the development of small-molecule organic dyes is necessary preceding the widespread use of this imaging technique. Inorganic nanomaterials are excellent for fluorescence-based preclinical animal imaging and while many current nanomaterial NIR-II probes show strong promise for clinical translation, organic NIR-II dyes similar in structure and pharmacokinetics to the FDA approved NIR-I organic fluorophores such as ICG and methylene blue should expedite the transition of NIR-II imaging into the clinic^28^,^29^,^30^. Researchers without the specialized knowledge of nanomaterial-based fluorophores would benefit from organic NIR-II dyes that could be incorporated into bioimaging procedures currently relying on the plethora of visible and NIR-I dyes. CH-4T's complete aqueous solubility combined with its high serum quantum yield makes it an attractive NIR-II clinical candidate capable of being utilized with ease for biomedical vascular imaging procedures. CH-4T can dramatically reduce fluorescent dye doses by ∼25-fold for biomedical imaging compared to previous, low quantum yield contrast agents such as CH-PEG. CH-4T can also uniquely enable the fastest temporal resolution in the NIR-II spectral window

The D–A–D NIR-II dye architecture shows strong potential for developing an array of NIR-II fluorescent probes through the molecular engineering of their constituent components. The addition of the negatively charged sulfonate groups translated into a dramatic boost in the *in vivo* quantum yield of CH1055 that allowed for video-rate imaging at the fastest frame rate along with the lowest exposures time to date for imaging at NIR-II wavelengths^1^. Small changes in the molecular structure of CH1055 yielded a dramatic increase in the optical performance of the D–A–D dye, while further optimization of the electron acceptor, *π*-spacers, electron donors and functional groups is likely to further increase the maximum attainable NIR-II quantum yield.

The advent of a high quantum yield NIR-II fluorophore enables many exciting possibilities for NIR-II medical imaging in terms of exposure time, imaging speed and penetration depth. Carbon nanotubes, encapsulated organic dyes and Ag_2_S quantum dot contrast agents have required exposure times of 100 ms and may even exceed 200 ms for video-rate imaging^2^,^3^,^4^,^5^. The instrument overhead time for normal video-rate imaging is ∼80 ms and results in a frame rate of 5.3 FPS at a typical NIR-II exposure time of 100 ms. The donor–acceptor NIR-II polymer with a nominal 1.4% quantum yield allowed ultra-fast videos at ∼20 ms exposure times and frame rates of 25 FPS^1^. The CH-4T complex was able to easily capture ultra-fast videos under 1.5–2 ms exposure times at 50 FPS, which is virtually at the maximum possible frame rate of our imaging system (52 FPS) given the instrument overhead time of 19 ms under ultra-fast imaging conditions.

Even though CH-4T has a significant higher brightness than the NIR-II polymer, the achievable frame rate with CH-4T is only double that of the polymer as the instrument overhead time is now the main factor limiting the time in-between frames^1^. However, designing faster fluorescent NIR-II imaging systems through hardware and software modifications could theoretically yield frame rates surpassing 500 FPS given the 1.5–2 ms exposure time of the CH-4T complexes. Such a high-brightness dye additionally allows the detection of deeper features in the body as evidenced by the clear resolution of the lumbar lymph nodes. As the excitation power density drops off when penetrating deeper into the body, high quantum yield NIR-II fluorophores are necessary to generate sufficient fluorescent signal. Although previous materials such as carbon nanotubes or CH-PEG can clearly discern more shallow features with a high degree of clarity due to minimal scattering, they have an insufficient quantum yield to detect deep features (Supplementary Fig. 13). The high quantum yield NIR-II dye also lowers the requisite excitation power density as evidenced by the imaging of the lumbar lymph nodes with ICG and CH-4T/HSA-HT at an equivalent power density of 16 mW cm^−2^ and with similar exposure times. Previously, a 17.5-fold higher power density was required for imaging femoral blood vasculature in the hindlimb with carbon nanotubes in the NIR-II (808 nm excitation, 140 mW cm^−2^) compared to IR800 in the NIR-I (785 nm excitation, 8 mW cm^−2^)^4^.

At this juncture, we would like to highlight the variability in the reported absolute quantum yield of IR-26, the reference fluorophore that serves as the basis for all reported NIR-II quantum yields. The first reported quantum yield of IR-26 as 0.5% in 1,2-dichloroethane (DCE), the most widely applied value, has been called into question by recent reports placing the quantum at both 0.05 and 0.1% (refs 31, 32, 33, 34). The reported quantum yield for CH-4T and complexes is based on using HiPCO SWCNTs as a reference, whose quantum yield was similar to IR-26 (ref. 1). HiPCO SWCNTs were selected as a quantum yield reference as nanotubes are simple to disperse in water, demonstrate excellent stability unlike IR-26 that degrades in 1,2-dichloroethane over relatively short periods to time, and are the most common NIR-II contrast agent^5^. The brightness of NIR-II dyes will be listed relative to other NIR-II fluorophores as well as in absolute quantum yield ranges henceforth until the discrepancies in the reported quantum yield of IR-26 are resolved. As this work marks a transition in the reference absolute quantum yield, the absolute quantum yield of CH-4T is 1.1–11% with respect to IR-26=0.05–0.5%. Changing the absolute quantum yield of the reference only serves to scale the brightness of the entire NIR-II field and does not affect the relative brightness between NIR-II emitting materials. Regardless, CH-4T is by far the brightest organic fluorophore of any NIR-I or NIR-II dye emitting past 1,000 nm (Supplementary Fig. 14) with a 27-fold higher quantum yield than HiPCO SWCNTs, a classic NIR-II imaging probe.

Tuning the interactions between organic NIR-II dyes consisting of large, hydrophobic *π*-systems and plasma proteins is a route to brilliantly fluorescent dyes emitting past 1,000 nm. In a similar fashion to ICG's ∼4 × increase in brightness when exposed to serum proteins, the 110-fold fluorescence increase between CH-4T/FBS-HT and CH-4T in PBS most likely occurs through the combination of multiple processes^35^,^36^. First, the amphiphilic character of CH-4T may form aggregates in aqueous solution through van der Waals forces that reduce brightness through self-quenching which is known to occur in ICG^29^. The dispersion of aggregates by CH-4T individualization through protein binding should contribute to the serum boost in quantum yield. Second, CH-4T geometrical confinement by hydrophobic pockets should promote a rigid dye conformation that minimizes the torsional rotations that result in non-radiative decay^12^. Rigid molecules have shorter fluorescent lifetimes and generally higher quantum yields as they dissipate less energy though internal conversion compared to those with freely rotating parts^37^. Internal conversion is the most important competing non-emissive process for low energy-gap NIR-II dyes thus increasing rigidity through protein binding will favourably increase quantum yield. Heating magnifies these effects as CH-4T presumably becoming entangled in a rigid conformation as increased thermal energy allows access to typically inaccessible hydrophobic pockets. At higher temperatures, CH-4T may become completely encased in hydrophobic domains as proteins re-fold around the dye after heating to minimize the number of hydrophobic side-chains exposed to water. In addition, donor–acceptor dyes are subject to twisted intramolecular charge transfer (TICT) in polar environments, especially if comprised of strong electron donors and acceptors^38^,^39^,^40^,^41^. Molecular simulations predict the core of CH1055 can exist in non-planar conformations that may contribute to a red-shifted TICT conformation that can dampen fluorescence^12^. The observed hypsochromic shift of ∼50 nm can be explained through CH-4T adopting a planar configuration and the alignment of *p*-orbitals when binding to proteins via hydrophobic or charge interactions in conjunction with minimizing solvent relaxation.

Developing a fundamental understanding of the manner in which NIR-II organic dyes interact with biological tissue is of critical importance for creating probes with high SBR ratios as well as high excretion levels. CH-4T demonstrated an impressively high quantum yield thorough protein binding and the dye was excreted through the biliary system with little residual dye left in normal tissue just days post-injection. Unlike current NIR-II nanomaterial fluorophores, CH-4T demonstrated complete excretion from the body, albeit at a slower rate than CH-PEG (Supplementary Fig. 15). CH-4T elicited no observable cellular toxicity (Supplementary Figs 16 and 17), while CH-4T/FBS-HT and CH-4T/HSA-HT showed a marginal reduction in cell viability at higher concentrations (Supplementary Figs 18 and 19). However, in a similar manner to the pioneering work by Frangioni and colleagues, tailoring the interaction strength between NIR-II fluorophores and proteins to maintain a high quantum yield while employing strategies such as polyionic yet net-neutral probes may expedite excretion kinetics and reduce non-specific *in vivo* binding^42^,^43^. The same rational design principles developed for NIR-I fluorophores may be applied to NIR-II organic dyes to develop the next generation of contrast and molecular imaging agents.

Hemodynamic imaging with a high frame rate in the NIR-II poses several advantages over other vascular flow imaging modalities such as laser speckle flowgraphy and photoacoustic imaging (PI). Laser speckle flowgraphy computes blood velocity based on the measurement of laser speckle blurring (termed normalize blur (NB)) from coherent light scattering from diffusing sources^44^,^45^. Modulation of the laser backscattered interference pattern occurs as red blood cells travel through capillaries. Laser speckle flowgraphy has significant clinical potential as it requires no exogenous contrast agents yet this imaging modality currently suffers from a number limitations. Measurement of laser flux depends highly on tissue properties that can vary from person to person or in disparate disease states. A correlation between the unit-less NB and capillary blood flow velocities occurs only under a narrow set of conditions^44^,^45^. This disallows facile blood velocity comparisons among people or even between an individual's eyes during retinal vasculature imaging. Photoacoustic imaging can additionally measure blood velocity without requiring exogenous contrast agents by exploiting hemoglobin's strong visible absorbance. However, with minimum resolvable features on the scale of ∼10–100 μm, photoacoustic imaging cannot compete with fluorescence imaging in terms of spatial resolutionx^46^. CH-4T's penetration depth (1–3 mm) combined with high temporal (50 FPS) and spatial (∼1 μm) resolution enables high-fidelity hemodynamic imaging of vessel sizes ranging from microvasculature to major vessels. Application of high frame-rate NIR-II imaging with CH-4T for hemodynamic visualization within tumours, intravascular tracking of circulating tumour cells or post-trauma cerebral microvascular imaging may provide key insight into disease pathophysiology. In addition to providing absolute blood velocities, CH-4T's high-brightness and long-wavelength emission may improve other biological optical imaging modalities. For instance, one-photon confocal imaging with CH-4T may push the penetration depth limit past that achievable with the current palette of visible/NIR-I/NIR-II fluorophores.

Even with the superior NIR-I silicon CCD detector specifications (for example, pixel number, dark current, readout noise and so on), NIR-II imaging on the InGaAs detector with longer wavelength probes improves imaging metrics. As no current silicon detector has suitable sensitivity past ∼1,000 nm, biomedical fluorescent imaging in the NIR-II depended on the emergence of InGaAs focal plane array detectors. However, similarly to the benefits garnered by pushing probe emission out of the visible (∼350–600 nm) and into the NIR-I (∼650–900 nm) on Si cameras, the development of bright, long-wavelength NIR-II probes (>1,000 nm) boosts imaging performance metrics. Internal comparisons within a broad wavelength range on an InGaAs detector have dramatically illustrated the imaging improvements by developing longer wavelength probes from 850 nm (ICG) to 1,100 nm (HiPCO SWCNTs) to 1,500 nm (laser vaporization SWCNTs)^14^. ICG and laser vaporization SWCNTs both emit in a region with similarly reduced InGaAs quantum efficiency of ∼30–40% compared to ∼85% at ∼1,100 nm with HiPCO SWCNTs. This indicates the marked differences in imaging quality at progressively longer wavelengths arise from reduced scattering/autofluorescence rather than variable detector spectral sensitivities. Fundamental advances in fluorophore chemistry have largely driven the NIR-II imaging field, making InGaAs detectors more commonplace for biomedical imaging. While newer generations of InGaAs detectors can uniformly increase sensitivity/SBR of all NIR-II probes, relative imaging metrics within the NIR-II spectral range will still be governed by wavelength dependent light-tissue interactions. Improving NIR-II fluorescent probes (that is, increasing both quantum yield and NIR-II emission wavelength) should proceed in parallel with the ongoing innovations in improving InGaAs cameras.

In summary, we have developed the first NIR-II contrast agent based on a small-molecule organic dye with an ultra-high quantum yield of 1.1–11% (IR-26=0.05–0.5%). The negatively charged sulfonate groups enabled supramolecular binding to serum proteins capable of a 110-fold fluorescence enhancement compared to CH-4T in PBS. Heating the CH-4T–protein complex caused a marked boost in fluorescence intensity indicating that brightness increases are possible through optimizing dye–protein interactions. *In vivo* vascular imaging was performed at 50 FPS, the fastest frame rate to date in the NIR-II at an exposure time of 2 ms to unambiguously resolve vascular hemodynamics. In addition, imaging of deep lymph nodes at a depth of ∼5–8 mm in both the first and second NIR windows clearly demonstrated the benefits of imaging deep anatomical features at longer wavelengths. CH-4T illustrates for the first time that high quantum yield dyes in the NIR-II are possible. Future work will strive to modify CH-4T to enable covalent linkable NIR-II fluorophores whose brightness can be utilized for ultra-high SBR *in vivo* molecular imaging.

## Methods

### Animal handling

All vertebrate animal experiments were performed under the approval of Stanford University's Administrative Panel on Laboratory Animal Care. Eight-week-old female NU/NU mice were obtained from Charles River for all imaging studies and housed at the Research Animal Facility of Stanford under our approved animal protocols. Before vessel or lymphatic imaging, all mice were anaesthetized in a rodent anaesthesia machine with 2 l min^−1^ O_2_ gas mixed with 3% isoflurane. Tail vein injection of contrast agents were carried with a catheter and synchronized with a camera that started continuous image acquisition simultaneously. The injected dose was a 200–300 μl bolus in a 1 × PBS solution at specified concentrations. During the time course of imaging the mouse was kept anaesthetized by a nose cone delivering 2 l min^−1^ O_2_ gas mixed with 3% isoflurane. The sample sizes of mice were selected based on previously reported studies. Mice were randomly selected from cages for all experiments. No blinding was performed. All groups within study contained *n*=3 mice.

### Synthesis of CH-4T

Dissolve 1 mg CH1055 (1.03 μmol) in 100 μl of dry DMSO. Then 5 mg taurine (40 μmol), 7 μl DIPEA (5.17 mg, 40 μmol) were added. Stirred for 2 min and then add HBTU 5 mg (13.2 μmol). The reaction solution was stirred overnight at room temperature under a nitrogen atmosphere. After the reaction finished, 100 μl of water was added and stirred for 1 hr to quench excess HBTU. Finally, Dionex Summit high-performance liquid chromatography (HPLC) system (Dionex Corporation, Sunnyvale, CA, USA), 340U four-channel ultraviolet-visible absorbance detector, Dionex C4, 9.4 mm × 250 mm semi-preparative column, gradient elution starting from 5% acetonitrile and ending up with 95% acetonitrile (in water with 0.1% TFA) at 42 min, 3 ml min^−1^ flow rate, 254 nm and 650 nm detection wavelength was used to purify the reaction. Overall, 1.3 mg CH1055-4Taurine (yield 93%) was produced as a green solid. MALDI-TOF-MS was used to identify the product. MALDI-TOF-MS calculated for: [C_62_H_64_N_10_O_16_S_6_] (M.W.): 1,396.3. Found: 1,396.2. See Supplementary Information for additional details.

### DGU of CH-4T and CH-4T complexes

Sucrose density gradients were prepared in 4 ml centrifuge tube. A total of 600 μl of 55, 50, 45, 40, 35, 30% w/w sucrose in 1 × PBS were added through a syringe with a needled pressed against centrifuge tube wall to not cause layer mixing. After adding all density layers, the centrifuge tube was placed at 10° from horizontal to allow for interlayer diffusion to smooth out density steps. After 1 h, CH-4T samples were loaded on top of the gradient. Any excess space in centrifuge tube was filled with 1 × PBS until meniscus reached ∼3 mm from top of centrifuge tube. The gradient was run in an ultracentrifuge (Beckman Coulter, Optima L-90K) using a Ti-55 rotor at 50,000 r.p.m. for 50 h.

### Preparation of CH-4T heated complexes

Prepared 784 μM CH-4T, 78.4 μM HSA/BSA in 1 × PBS solution. Added 10 μl of 784 μM CH-4T into 200 μl of 78.4 μM HSA/BSA solution to keep the molar ratio of CH-4T:HSA at 1:2 for optical characterization (1:1 ratio for *in vivo* HSA imaging). (HSA: Sigma Aldrich, Lot# SLBN5035V, SLBL6442V; BSA: Sigma Aldrich, Lot# SLBR6762V). Keep overall protein concentration as low as possible to prevent possible gelling after heating. For FBS, use maximum dye loading of 50 μg CH-4T/100 μl FBS (see Supplementary Fig. 4 for manufacturer/batches). Vortex the solution to mix evenly. Then add the dye–protein complex in a sealed eppendorf into different temperature water baths for 10 min (∼70–75 °C for *in vivo* complexes). Discard if solution gels or denatures by turning white. Temperatures kept under 80 °C should not gel. If not used within a few hours, store the heated dye–protein solution at 4 °C for long-term storage. CH-PEG heated complexes were prepared by the same procedure for comparison.

### Spectral characterization of CH-4T

Absorbance spectra of CH-4T and complexes were taken on a ultraviolet-visible-NIR Cary 6000i spectrometer that was background corrected for each biological media such as water, FBS and HSA protein solutions. The NIR-II fluorescence emission spectrum was captured on a home-built spectroscopy set-up by exciting CH-4T and complexes with an 808 nm laser diode with a power output of ∼160 mW. The excitation laser was filtered with a combination of an 850 (Thorlabs)/1,000 (Thorlabs)/1,100 (Omega)/1,200 (Thorlabs)/1,300 (Omega) nm short-pass filters. Samples were added to either a 1 mm or 1 cm path-length cuvette and the resulting emission filtered through a 910 nm long-pass filter (Thorlabs) to reject the incident excitation laser light. The emitted fluorescence was collected on a spectrometer (Acton SP2300i) coupled to a linear liquid nitrogen cooled InGaAs detector array (Princeton Instruments, OMA-V). After collecting the raw acquisition data, a correction file was applied to correct for the variable InGaAs quantum efficiency as a function of detection wavelength as well as the variable 910 nm long-pass filter extinction features across the NIR-II spectral region. Fluorescence enhancement defied as: 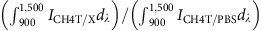, where *I*_CH−4T/X_ corresponds to the fluorescent emission spectrum of protein-bound CH-4T. All fluorescent enhancement values were derived from measurements on the wavelength-corrected photospectrometer unless specifically stated otherwise.

### Measuring NIR-II quantum yield

The fluorescence quantum yield of CH-4T and complexes were measured in a similar manner as described in previous publications^1^,^2^. Briefly, HiPCO SWCNTs were utilized as a reference fluorophore due to their high aqueous stability. The quantum yield of HiPCO SWCNTs has previously been determined as 0.4% based on an IR-26 quantum yield of 0.5% in DCE (please see Discussion for further details on IR-26 quantum yield). A serial dilution of five solutions of HiPCO SWCNTs as well as CH-4T and complexes with an OD <0.1 at 808 nm was measured to confirm absorbance values at 808 nm and the fluorescent emission spectrum was collected on a wavelength-corrected NIR-II spectrometer in a 1 cm quartz cuvette (Starna) in the manner specified above. The fluorescent emission spectrum was integrated and plotted against the OD value at 808 nm and a linear fit was applied to verify the linearity between fluorescent brightness and concentration. For the brightest samples, inter-filter effects were seen ∼OD 0.1 at 808 nm, thus lower concentration ranges were utilized. By comparing the slope of the linear fit between HiPCO SWCNTs and CH-4T and complexes, the quantum yield was determined based on the following supporting equation (1):


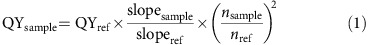


where QY_sample_ is the QY of CH-4T and complexes, QY_ref_ is the QY of HiPCO SWCNTs in water (0.4% based on IR-26=0.5% in DCE^1^), *n*_sample_ and *n*_ref_ are the refractive indices of HiPCO SWCNTs, CH-4T and complex solutions which are both water (1.33) in this case.

### Video-rate and ultra-fast NIR-II imaging

Video-rate and ultra-fast NIR-II imaging was carried out in a similar manner as previous publication^1^,^2^. Briefly, all NIR-II images were collected on a 319 × 256 pixel two-dimensional InGaAs array (Princeton Instruments 2D OMA-V 1.7; pixel number: 319 × 256; readout noise: 50 electron r.m.s.; dark signal: 5,000 electrons per second per pixel) utilizing a home-built imaging set-up. The excitation was provided by an 808 nm diode laser (RMPPC Lasers) through an optical fibre and collimator at a power density of 140 mW cm^−2^. The 808 nm laser was filtered with 850/1,000 nm short-pass filters (Thorlabs). Fluorescence emission was collected with a 910 nm long-pass filter (Thorlabs) combined with either a 1,000 nm long-pass filter (Thorlabs) or 1,100 nm long-pass filter (Thorlabs) as specified. A ‘ × 2.5' magnification lens set (‘C' coated, 200 mm and 75 mm, Thorlabs) was used for the mice hindlimb NIR-II imaging. Changing the relative position of the two NIR achromats can modify the magnifications into ‘ × 1' for whole body imaging. For ultra-fast imaging, the camera is set to continuously expose using LabView software with a 19-ms overhead time caused by camera readout. Images and videos were processed with MATLAB. Imaging the mouse in a supine position with the leg and chest region well secured on the imaging platform with medical tape prevented any motion artifacts resulting from breathing motion or heartbeat. Selecting a 3 × 3 pixel region corresponding to the femoral artery in each video frame allowed for analysis of the fluorescent intensity in hindlimb vasculature. The femoral vasculature mapped to the same pixel group throughout imaging, indicating that the leg is secure and that the periodic patterns do not arise from breathing induced artifacts. Please see Supplementary Sections 12 and 13 for detailed schematic of optical path with component product information and catalogue numbers.

### Sacral/popliteal and lumbar lymph node NIR-I/II imaging

Sacral/popliteal and lumbar lymph node NIR- II imaging was performed using the whole body set-up as described in the above paragraph. A 1,344 × 1,024-pixel Hamamatsu silicon CCD detector (Hamamatsu C8484-03G02; pixel number: 1,344 × 1,024; readout noise: ∼7 electron r.m.s.; dark current: 0.01 electrons per second per pixel) with a 785 nm long-pass filter (Semrock) for NIR-I imaging was added in parallel to the NIR-II set-up for NIR-I imaging comparisons as described in previous publications^4^. The 785 nm laser (filtered with 780 nm band-pass filter) was used both for NIR-I and NIR-II imaging with a power density of 16 mW cm^−2^. All contrast agents (ICG, CH-4T, CH-4T/HSA-HT) were injected into each digit and the footpad under anaesthesia. Mice were then immediately placed on the imaging platform. A gentle massage was performed on the injection site for faster diffusion into the lymphatic system. For deep lumbar lymph node imaging, ICG was first injected as described above. The mice were taped securely in a position where the lumbar lymph nodes were visualized on the silicon NIR-I detector. CH-4T complexes were subsequently injected ∼10 min later for imaging in the NIR-II. Fluorescent intensity cross-sectional profiles in the NIR-I versus NIR-II were analysed in Image J. Please see Supplementary Sections 12 and 13 for detailed schematic of optical path with component product information and catalogue numbers.

### Data availability

Data supporting the findings of this study are available within the article (and its Supplementary Information files) and from the corresponding author upon reasonable request.

## Additional information

**How to cite this article:** Antaris, A. L. *et al*. A high quantum yield molecule-protein complex fluorophore for near-infrared II imaging. *Nat. Commun.* **8,** 15269 doi: 10.1038/ncomms15269 (2017).

**Publisher's note**: Springer Nature remains neutral with regard to jurisdictional claims in published maps and institutional affiliations.

## Supplementary Material

Supplementary Information Supplementary Figures and Supplementary Methods.
