# Supplementary material for: A high quantum yield molecule-protein complex fluorophore for near-infrared II imaging
Source: Nat Commun. 2017 May 19;8:15269. doi: 10.1038/ncomms15269 (PMC5454457; doi:10.1038/ncomms15269)
Supplement: Supplementary Information — Supplementary Figures and Supplementary Methods. [file ncomms15269-s1.pdf]

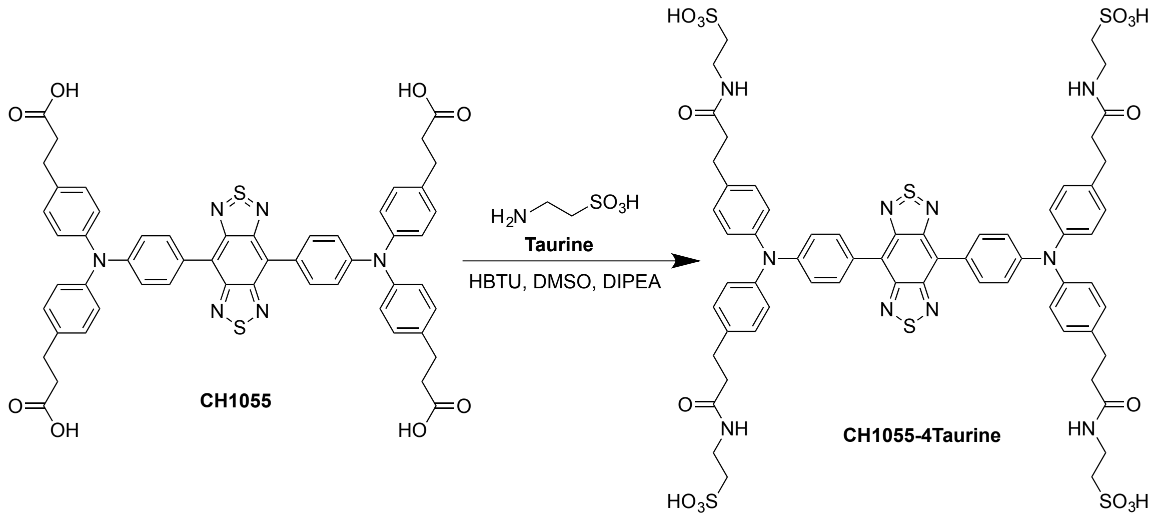

Supplementary Methods: One step synthesis of CH-4T from CH1055

Synthesis details:
Dissolved 1mg CH1055 (1.03 μmol) in 100μL dry DMSO. Then added 5mg taurine (40 μmol), 7μL DIPEA (5.17mg, 40 μmol). Stirred for 2 mins and then added HBTU 5mg (13.2 μmol). The reaction solution was stirred over night at room temperature under a nitrogen atmosphere. After the reaction finished, 100 μL of water was added and stirred for 1h to quench the excess HBTU. Finally, Dionex Summit high-performance liquid chromatography (HPLC) system (Dionex Corporation, Sunnyvale, CA), 340U four-channel UV-Vis absorbance detector, Dionex C4, 9.4 mm × 250 mm semi-preparative column, gradient elution starting from 5% acetonitrile and ending up with 95% acetonitrile (in water with 0.1% TFA) at 42 mins, 3 mL/min flow rate, 254 nm and 650 nm detection wavelength was used to purify the reaction. 1.3 mg CH1055-4Taurine (Yield 93%) was got as a green sold. MALDI-TOF-MS was used to identify the product. MALDI-TOF-MS Calcd for: [$C_{62}H_{64}N_{10}O_{16}S_6$] (M.W.): 1396.3. Found: 1396.2.

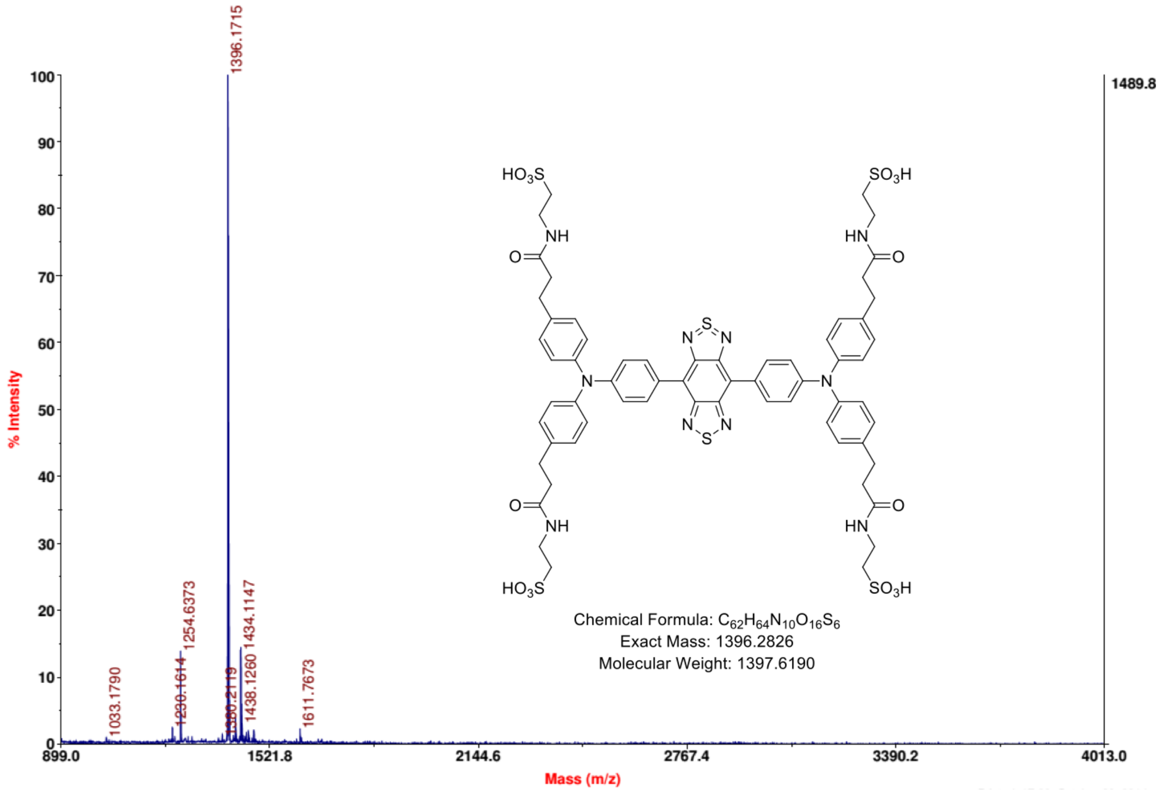

Supplementary Figure 1: MALDI-TOF-MS of CH-4T. Molecular weight of [$C_{62}H_{64}N_{10}O_{16}S_6$] calculated to be 1396.3 and MALDI-TOF-MS analysis of the product resulted in a molecular weight of 1396.2. AB SCIEX 5800 TOF/TOF MALDI Mass spectrometer System, CHCA as matrix, reflector mode was chosen.

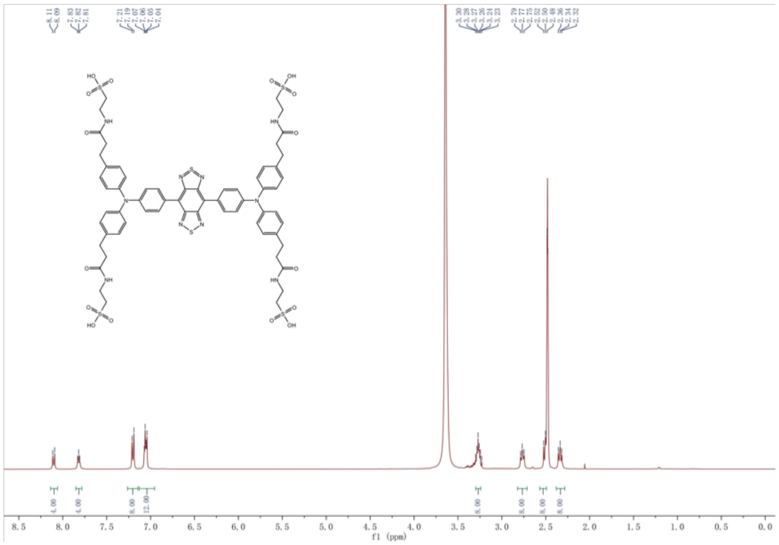

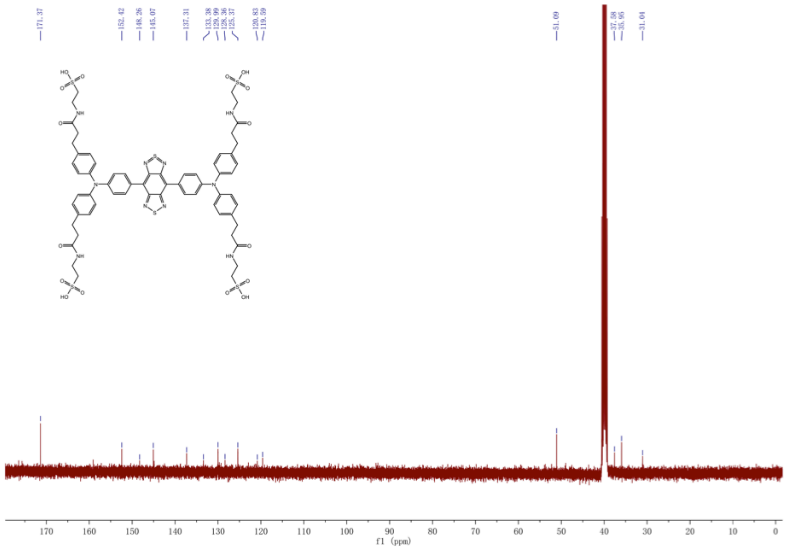

Supplementary Figure 2: NMR of CH-4T

^{1}H NMR (400 MHz, DMSO) δ 8.10 (d, *J* = 8.7 Hz, 4H), 7.82 (t, *J* = 5.4 Hz, 4H), 7.20 (d, *J* = 8.4 Hz, 8H), 7.06 (dd, *J* = 8.4, 3.9 Hz, 12H), 3.26 (dd, *J* = 10.6, 5.1 Hz, 8H), 2.82 – 2.71 (m, 8H), 2.51 (d, *J* = 7.9 Hz, 8H), 2.38 – 2.28 (m, 8H). ^{13}C NMR (101 MHz, DMSO) δ 171.37, 152.42, 148.26, 145.07, 137.31, 133.38, 129.99, 128.36, 125.37, 120.83, 119.59, 51.09, 37.58, 35.95, 31.04.

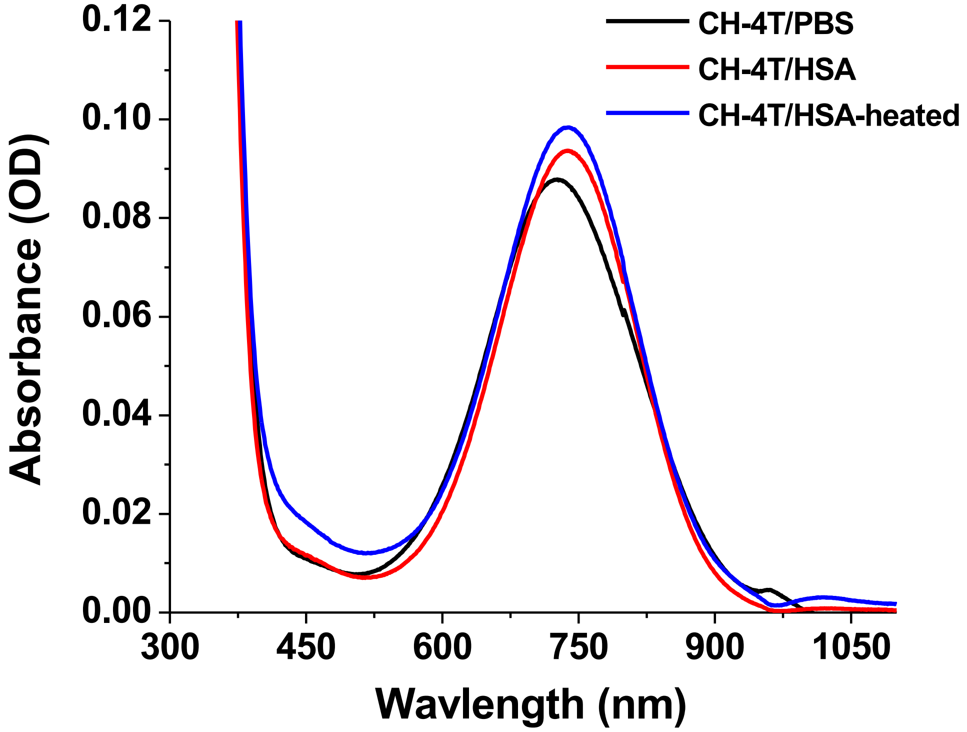

Supplementary Figure 3: UV-VIS of CH-4T in various media. Absorbance (OD) of CH-4T (10 μM) in PBS, HSA and HSA-heated (200 μM [HSA]). Absorbance peak of CH-4T at 738 nm which red-shifts to 748 nm after mixing with HSA. A slight increase in absorbance is noted after mixing with HSA and a subsequent further increase after thermal stabilization at 75 °C for 10 minutes.

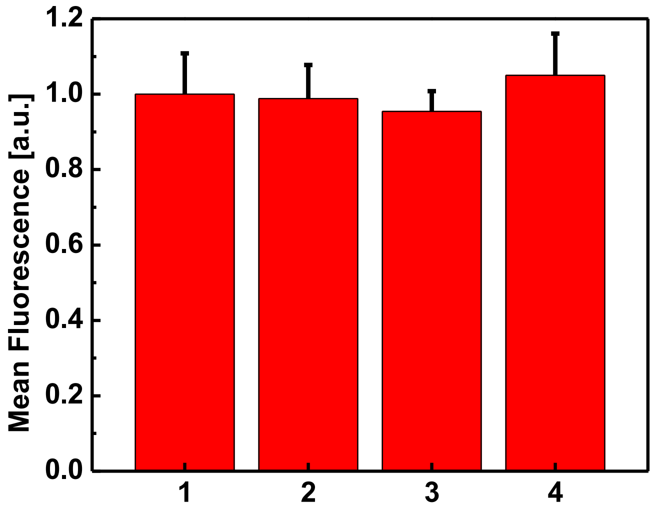

Supplementary Figure 4. Fluorescence intensity of CH-4T in FBS from various sources. (**1**) Hyclone FBS 40 nm filtered (batch# AYJ171940) (**2**) Hyclone FBS 0.1 µm filtered (batch# AZD188861) (**2**) Hyclone FBS triple 0.1 µm sterile filtered (AYF161496) (**4**) Axenia Biologix CultraPure FBS (batch# F001T0001). CH-4T at 3 µm; Images acquired at 20 ms exposure time with 900 long-pass filter. Error bars represent average ROI fluorescent intensity of three vials at equivalent CH-4T concentration.

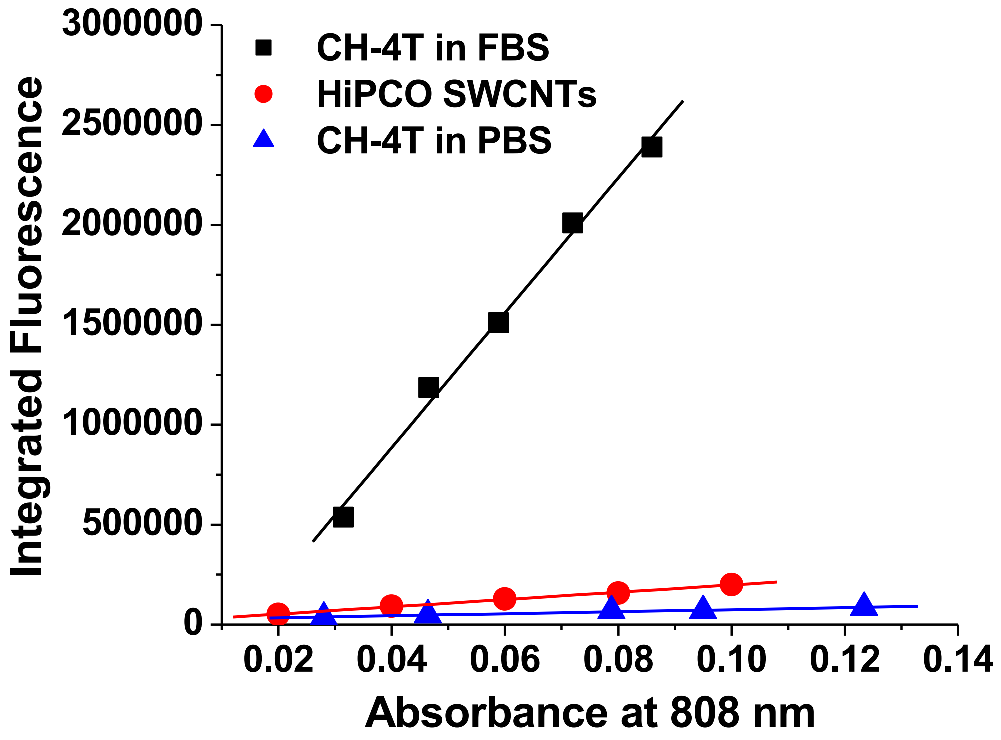

Supplementary Figure 5: Quantum yield of CH-4T in PBS. Plot of the integrated fluorescence spectrum of CH-4T in PBS and pre-mixed with FBS at five different concentrations (absorbance measured in OD). Linear fits were used to calculate quantum yield by comparing the slopes to reference HiPCO SWCNTs (QY = 0.4%).

$$QY_{CH-4T/PBS} = \frac{m_{CH-4T/PBS}}{m_{HiPCO}} * QY_{HiPCO} = 0.245 * 0.4\% = 0.098\% \quad (1)$$

Fluorophore	Quantum Yield (QY,%)	Relative QY
CH-4T/PBS	0.098	1
CH-4T/FBS	4.8	49
CH-4T/FBS-HT	10.8	110
CH-4T/HSA	2.4	24
CH-4T/HSA-HT	5.0	51

Supplementary Table 1: Absolute and relative quantum yield of CH-4T in various media. All absolute quantum yields taken with $QY_{IR\text{-}26}$ = 0.5% Relative quantum yield taken with respect to CH-4T/PBS.

Fluorophore	Quantum Yield (QY,%)	Relative QY
CH-4T/FBS-HT	10.8	1
HiPCO SWCNTs	0.4	0.037
CH-PEG	0.3	0.027
IR-26	0.5	0.046

Supplementary Table 2: Absolute and relative quantum yield of CH-4T compared to common NIR-II probes. All absolute quantum yields taken with QY_{IR-26} = 0.5% Relative quantum yield taken with respect to CH-4T/FBS-HT.

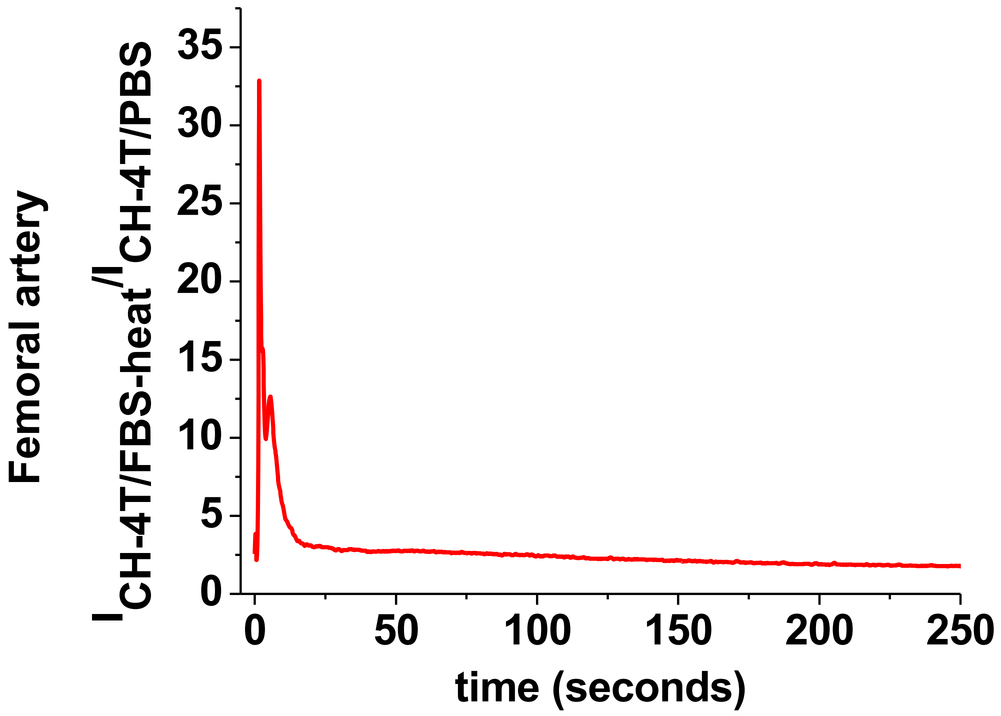

Supplementary Figure 6: Fluorescent intensity difference of CH-4T/FBS-heated versus free CH-4T. Integrated fluorescent intensity of an ROI region in the femoral artery of CH-4T/FBS-heated divided by free CH-4T during video-rate imaging (1000LP, 35 ms exposure time) in Figure 3d. The two observed peaks in above spectrum correspond to the presence of a peak in the CH-4T/FBS-heated spectrum in Figure 3d. The maximum difference in vessel intensity was ~33-fold during the first 10 seconds post-injection while the vessel intensity difference stabilized to a steady state value of ~2-fold at 200 seconds post-injection.

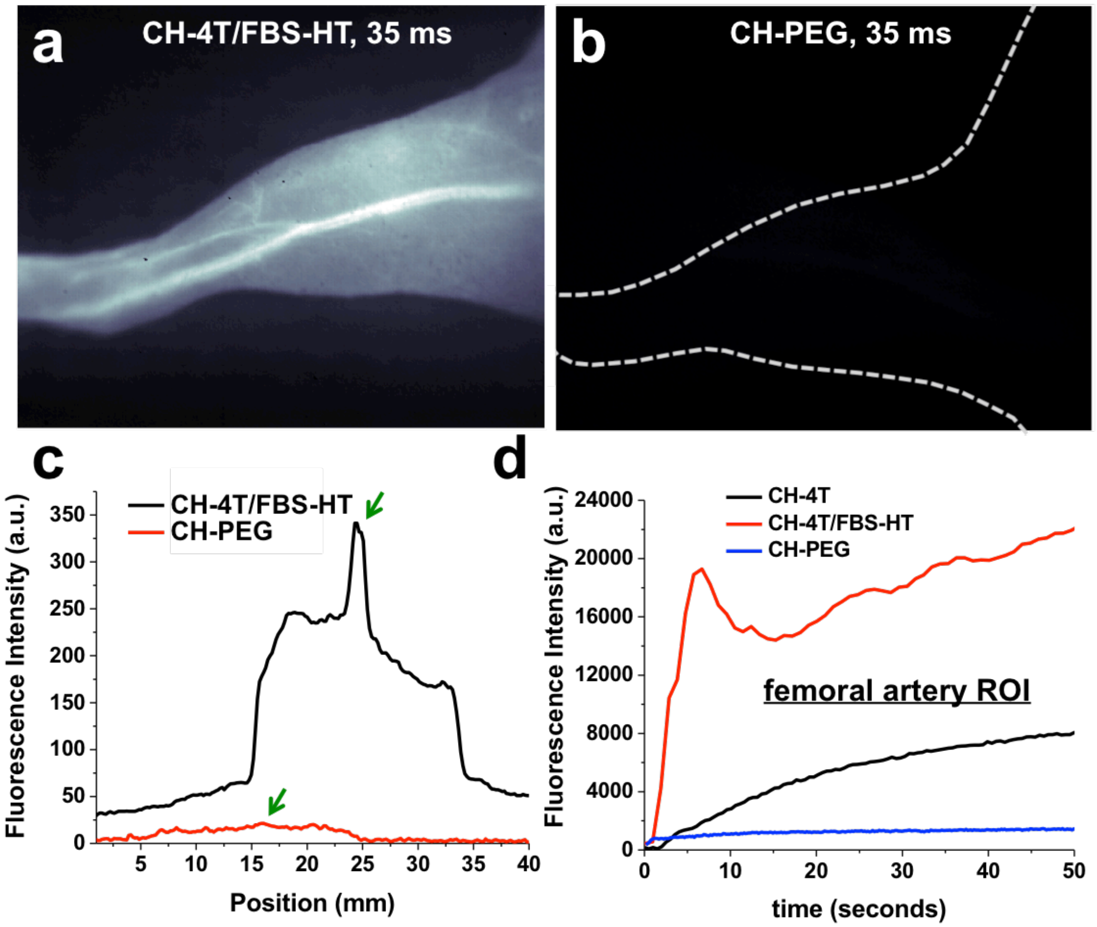

Supplementary Figure 7: Comparison of CH-4T/FBS-HT to CH1055-PEG (CH-PEG) for NIR-II vasculature imaging. NIR-II fluorescent images of mouse hindlimb vasculature ~10 minutes post-injection of **(a)** CH-4T/FBS-HT **(b)** and CH1055-PEG at equivalent dosages (OD 3.2 at 808 nm) and exposure times. (**c**) Cross-sectional fluorescent intensity profiles of mouse hindlimbs in (**a**,**b**) with green arrows marking position of femoral artery/vein. (**d**) Fluorescent intensity of integrated ROI region on the hindlimb femoral artery plotted for 50 seconds post-injection during video-rate imaging of CH-4T/FBS-HT, free CH-4T and CH1055-PEG (35 ms exposure time). All NIR-II imaging was performed with a 1000 nm long-pass emission filter.

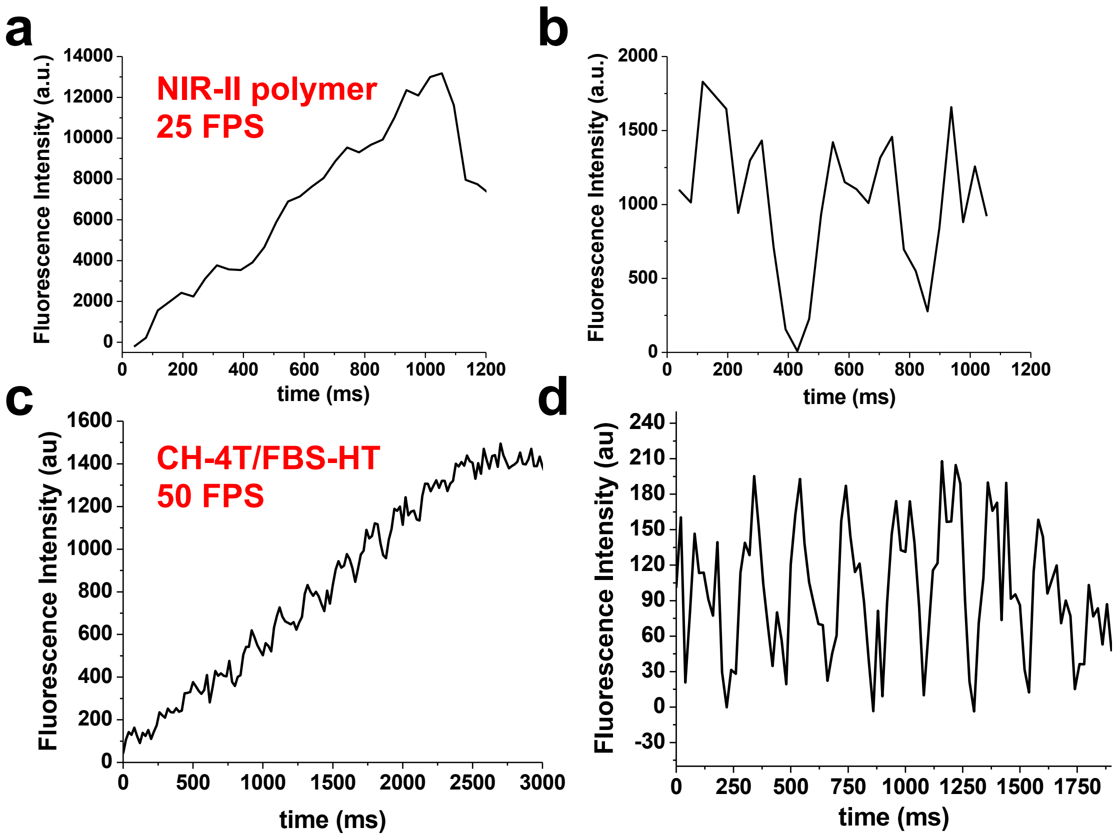

Supplementary Figure 8: CH-4T versus NIR-II polymer for cardiac cycle imaging. Cardiac cycle waveform derived from ROI analysis of mouse hindlimb femoral artery for (**a**) NIR-II polymer at 20 ms exposure time and 25 FPS and (**c**) CH-4T/FBS-HT at 2 ms exposure time and 50 FPS. Subtracting a linearly increasing background resolves superimposed periodic heartbeat pattern for (**b**) NIR-II polymer and (**c**) CH-4T/FBS-HT. All imaging performed with 1000 nm long-pass filter.

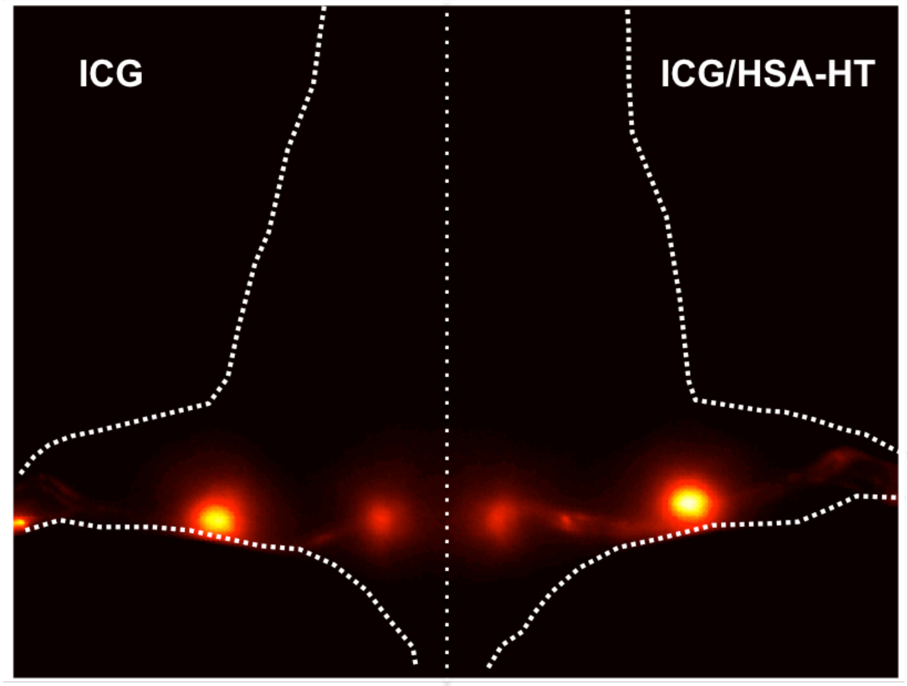

Supplementary Figure 9: Infrared lymph node imaging with ICG and ICG/HSA-HT. NIR-I fluorescent image ~30 minutes post-injection of 50 μM ICG (left), ICG/HSA-HT (right) (100 ms exposure). Image collected with 808 nm excitation laser (140 mW/cm^2) on InGaAs detector (850 nm long-pass filter). See Methods section for further experimental details.

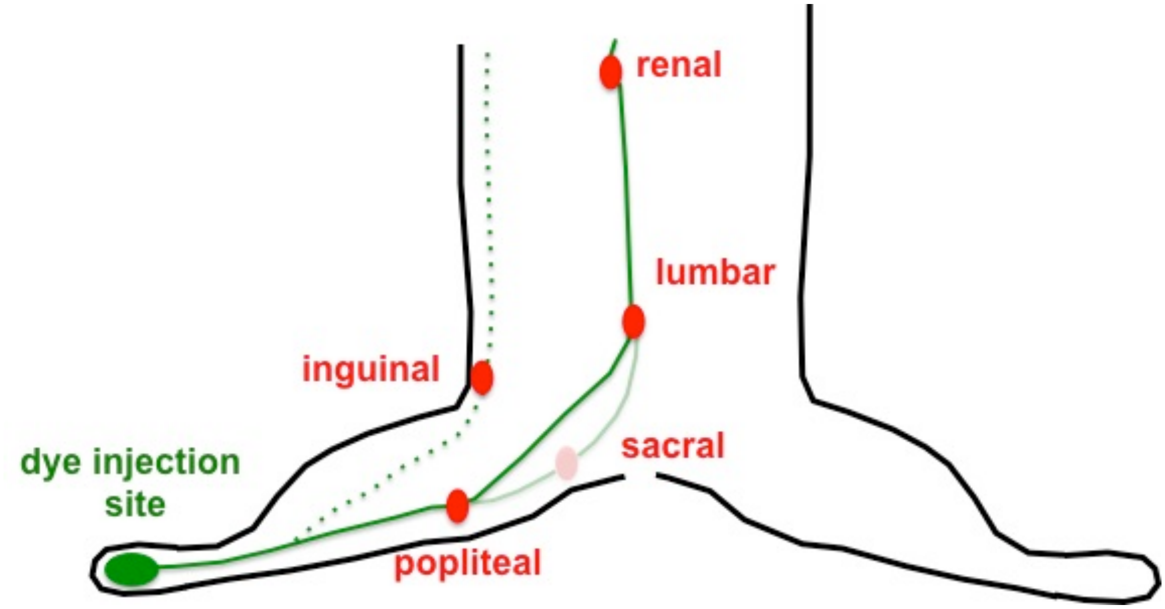

Supplementary Figure 10: Illustration of lymph nodes and vessels in supine mouse lower body. Fluorophore injection site on footpad and digits of the hindlimb shown as green circle. Vessels in main lymphatic drainage pathways depicted as solid green lines while secondary pathways depicted as dotted green line. Drainage from the foot region to the inguinal and subsequently the axillary lymph node occurs infrequently. Sacral lymph node shown as translucent as its only visible when mouse is in prone position.

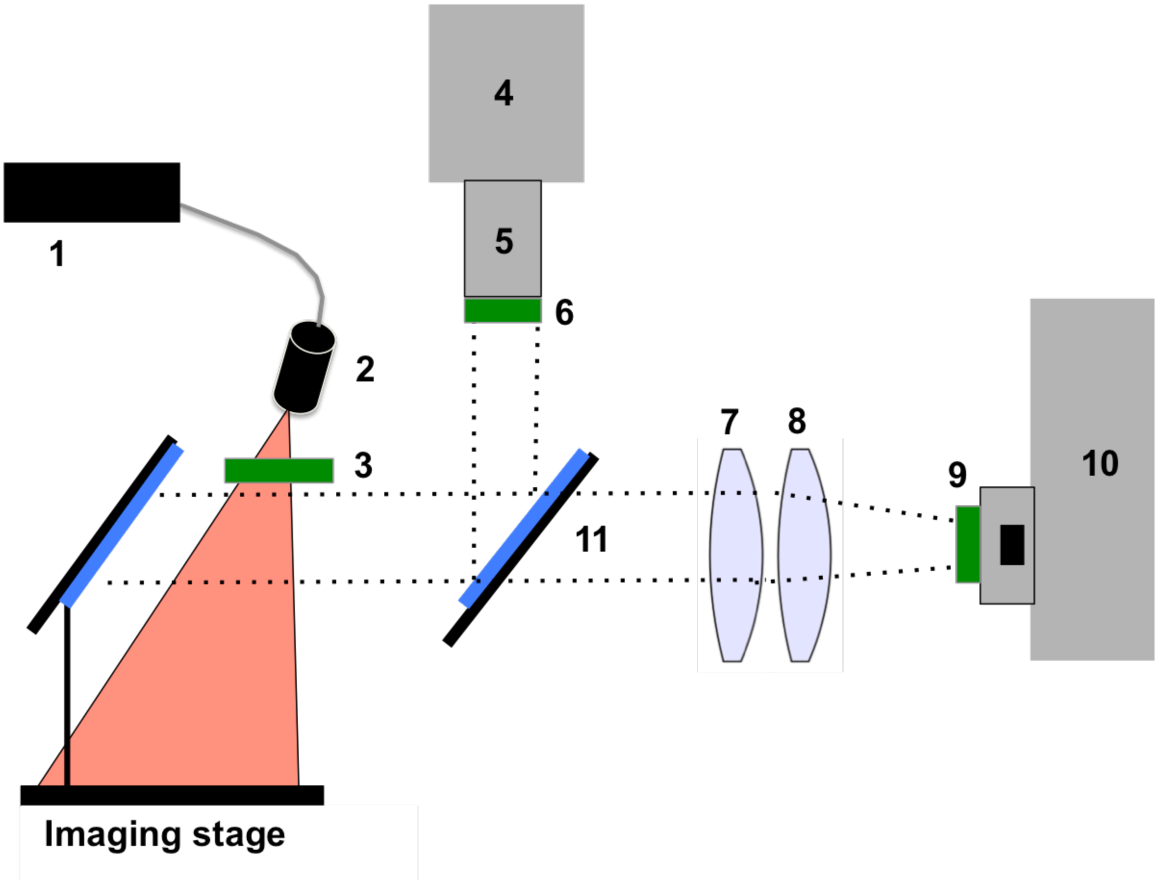

Supplementary Figure 11: Dual silicon (ICG), InGaAs (CH-4T) optical schematic. (**1**) 785 nm laser diode (CNI Laser, China). (**2**) 4.5 mm focal length collimator (Thorlabs; F230SMA-B). (**3**) 780 nm band-pass with 10 nm bandwidth (Thorlabs; FB780-10-01''). (**4**) silicon camera (Hamamatsu C8484-03G02) (**5**) telescope lens (Tamron 1:3.9 75 mm) (**6**) 785 long-pass filter (Semrock; BLP01-785R-25). (**7**) 200 mm NIR achromat (Thorlabs; AC508-200-C). (**8**) 75 mm NIR achromat (Thorlabs; AC508-75-C. (**9**) 910 long-pass and 1000 nm long-pass filter (Thorlabs; FEL-1000-01'', FEL-900-01''). (**10**) InGaAs camera (Princeton Instruments; OMA V 1.7. (**11**) flip-mirror.

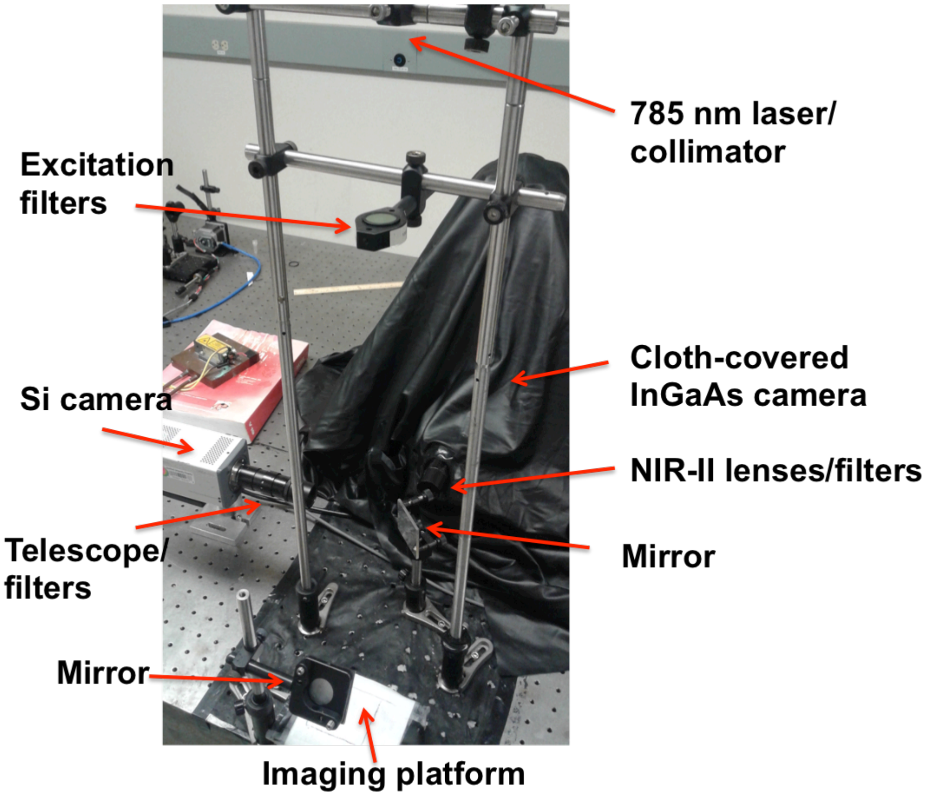

Supplementary Figure 12: Photograph of Si/InGaAs fluorescent imaging setup.

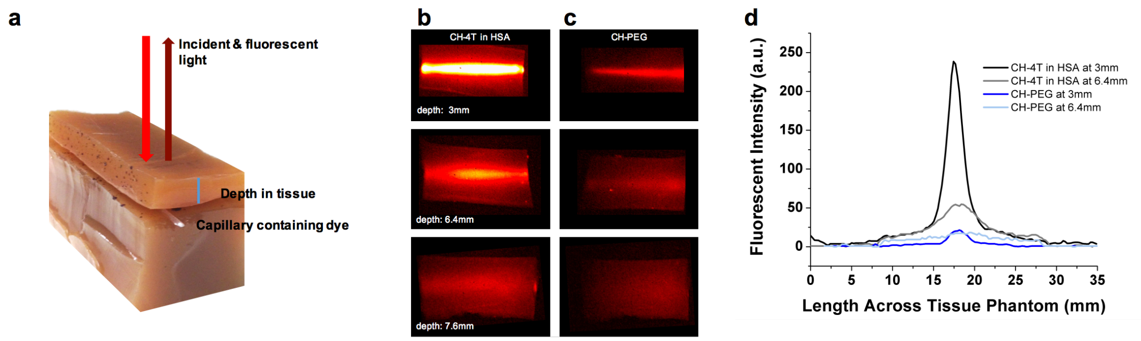

Supplementary Figure 13: NIR-II fluorescence of CH-4T/HSA and CH-PEG at different depths in tissue-mimicking phantom. (**a**) Experimental configuration showing dye placed in capillary tube under a layer of tissue phantom. (**b, c**) NIR-II fluorescent images (1050 nm long-pass) of capillary tube as tissue depth increases for CH- 4T/HSA and CH-PEG, respectively. (**d**) Fluorescent intensity cross-sectional profile of CH-4T and CH-PEG dyes at variable depths.

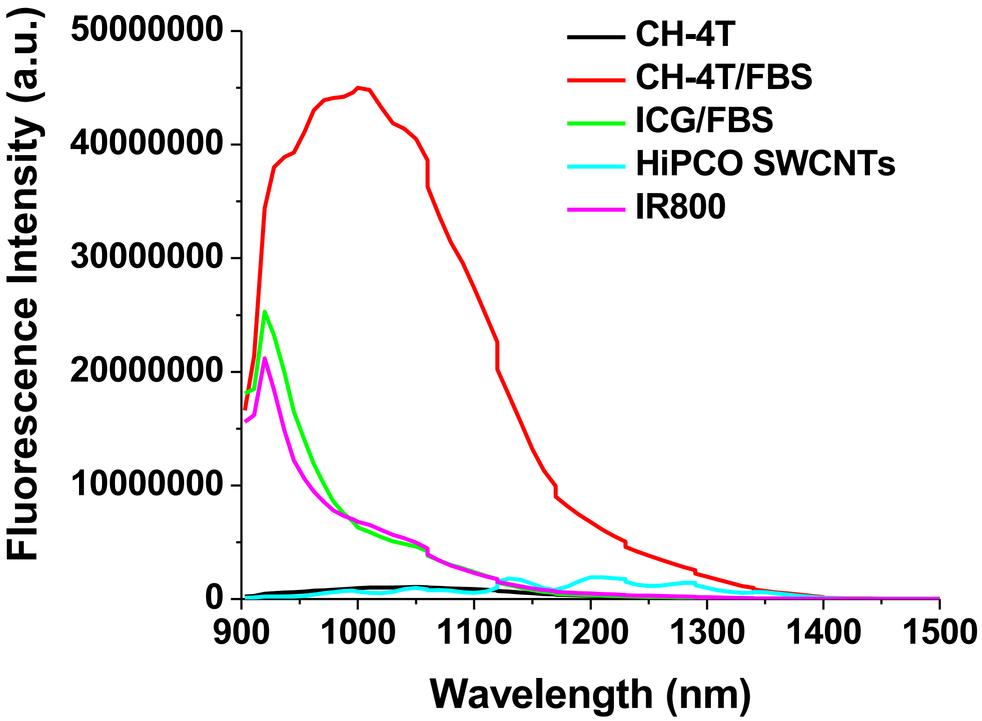

Supplementary Figure 14: Fluorescent emission of NIR-I/II dyes past 1000 nm. Emission spectrum at wavelengths longer than 900 nm after 808 nm laser excitation from NIR-II fluorophores including CH-4T, CH-4T/FBS and HiPCO carbon nanotubes as well as NIR-I dyes such as IR800 and ICG/FBS. ICG was added to FBS to maximize its fluorescent brightness.

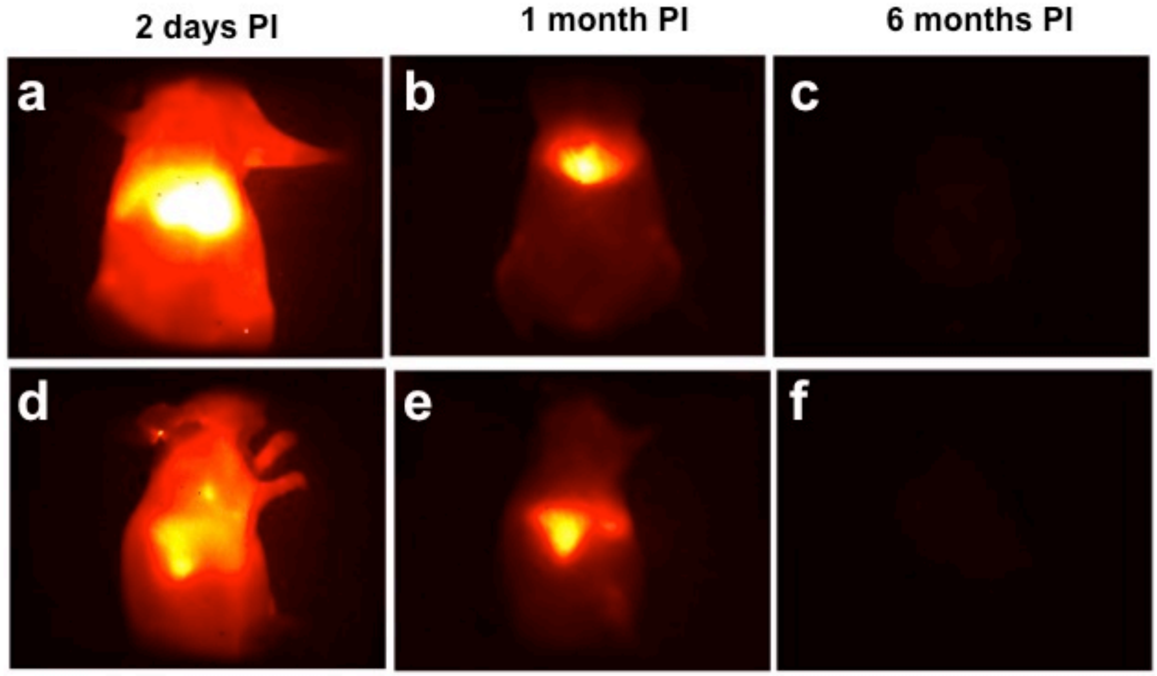

Supplementary Figure 15: NIR-II fluorescence monitoring of CH-4T whole-body clearance. Whole-body fluorescent images of mouse in (**a**) prone and (**c**) sagittal position 2 days post-injection and corresponding images (**b,d**) one month post-injection of 50 µg of CH-4T/PBS. Minimal uptake of CH-4T was observed in all organs and tissues other than the liver. All images taken with 1100 nm long-pass filter at an 80 ms exposure time.

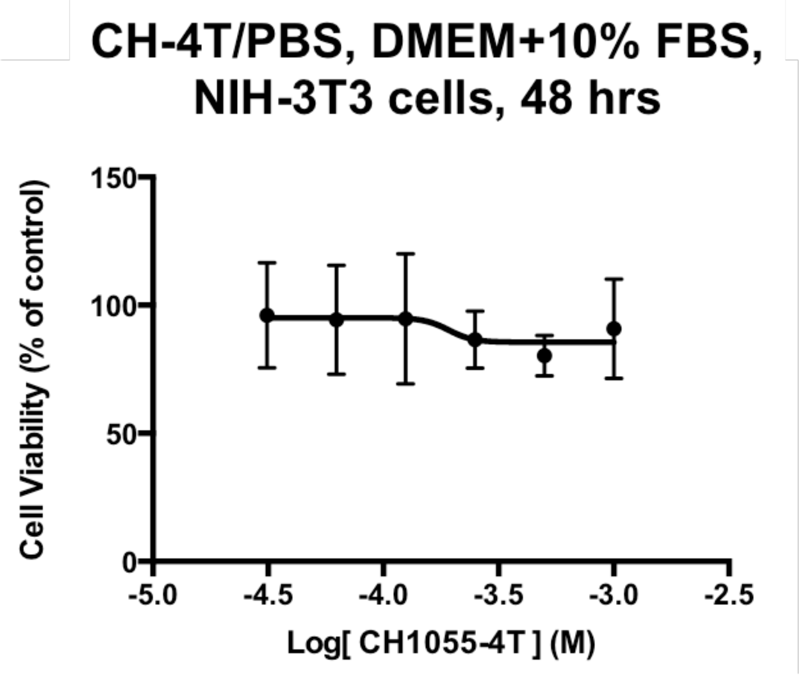

Supplementary Figure 16: Cell toxicity of CH-4T. Evaluated by using an MTT (3-(4,5- dimethylthiazol-2-yl)-2,5-diphenyl tetrazolium bromide) (Sigma-Aldrich, St. Louis, MO) assay. Murine fibroblast NIH-3T3 cells were plated into a 96 well plate at a concentration of $4*10^3$ cells/well in DMEM with 10% fetal bovine serum (FBS) at 37 ^oC and 5% CO^2, and 24 h later, the cells were incubated with CH-4T with different concentrations (1 mM, 500 μM, 250 μM, 125 μM, 62.5 μM, 31.25 μM, 0 μM) for 48 h. Old medium was taken out and new medium was added in 48 h later. Then 10 μL of the 12mM MTT stock solution was added and incubated for 3.5 hours. After removing medium, 150 μL dimethylsulfoxide was added to dissolve the formazan crystals precipitavvtes. After shaking the cell plate for 15 min, the absorbance(A) at a wavelength of 540 nm with a reference wavelength of 650 nm was measured with a Bio-Rad microplate reader. The relative cell viability (%) was calculated by $(A_{4T}/A_{blank}) \times 100$ (X values). The concentrations of 4T were calculated in log scales (Y values). Mean value and standard deviation were calculated using Graph Pad Prism version 7.0 (Graph Pad Software). Dose-response data table was chosen, with nonlinear regression the curve was gotten above. Error bars represent standard deviation. All samples were done in triplicate and the experiment was replicated three times.

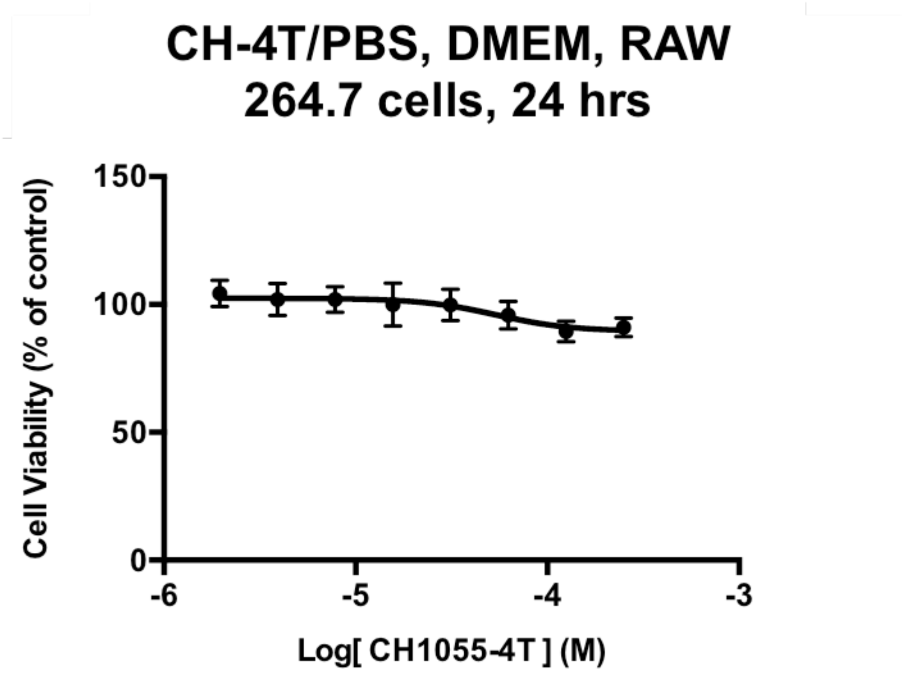

Supplementary Figure 17: Cell toxicity of CH-4T/PBS. Evaluated by using an MTT (3-(4,5- dimethylthiazol-2-yl)-2,5-diphenyl tetrazolium bromide) (Sigma-Aldrich, St. Louis, MO) assay. **Murine macrophage RAW 264.7 cells** plated into a 96 well plate at a concentration of $4*10^3$ cells/well in **DMEM with NO fetal bovine serum (FBS)** at 37 ^oC and 5% CO^2, and 24 h later, the cells were incubated with CH-4T with different concentrations (250 μM, 125 μM, 62.5 μM, 31.25 μM, 15.63 μM, 7.81 μM, 3.91 μM, 1.95 μM, 0.98 μM) for 24 h. . Old medium was taken out and new medium was added in 48 h later. Then 10 μL of the 12mM MTT stock solution was added and incubated for 3.5 hours. After removing medium, 150 μL dimethylsulfoxide was added to dissolve the formazan crystals precipitates. After shaking the cell plate for 15 min, the absorbance(A) at a wavelength of 540 nm with a reference wavelength of 650 nm was measured with a Bio-Rad microplate reader. The relative cell viability (%) was calculated by $(A_{4T}/A_{blank}) \times 100$ (X values). The concentrations of 4T were calculated in log scales (Y values). Mean value and standard deviation were calculated using Graph Pad Prism version 7.0 (Graph Pad Software). Dose-response data table was chosen, with nonlinear regression the curve was gotten above. Error bars represent standard deviation. All samples were done in triplicate and the experiment was replicated three times.

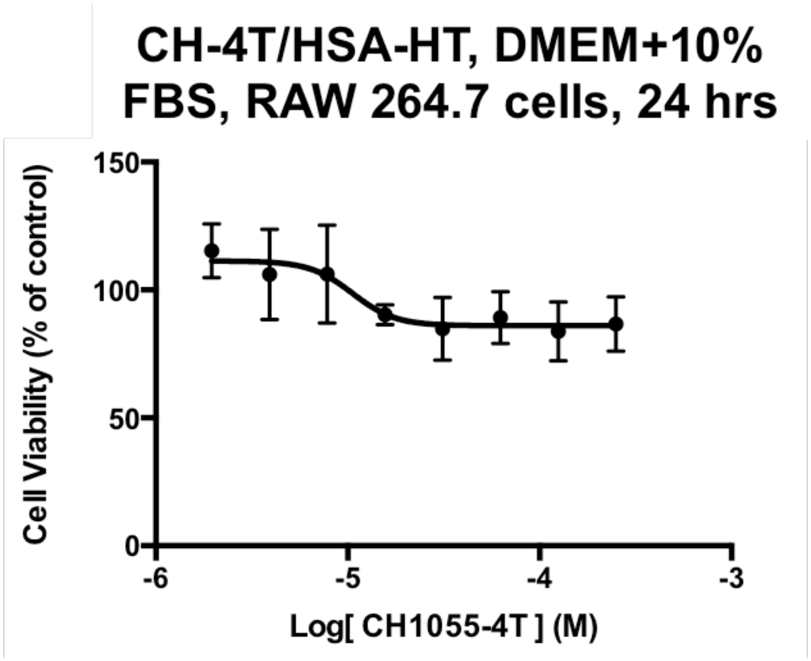

Supplementary Figure 18: Cell toxicity of CH-4T/HSA-HT. Evaluated by using an MTT (3-(4,5- dimethylthiazol-2-yl)-2,5-diphenyl tetrazolium bromide) (Sigma-Aldrich, St. Louis, MO) assay. **Murine macrophage RAW 264.7 cells** plated into a 96 well plate at a concentration of $4*10^3$ cells/well in **DMEM with 10% fetal bovine serum (FBS)** at 37 ^{o}C and 5% CO^2, and 24 h later, the cells were incubated with CH-4T with different concentrations (250 μM, 125 μM, 62.5 μM, 31.25 μM, 15.63 μM, 7.81 μM, 3.91 μM, 1.95 μM, 0.98 μM) for 24 h. Old medium was taken out and new medium was added in 48 h later. Then 10 μL of the 12mM MTT stock solution was added and incubated for 3.5 hours. After removing medium, 150 μL dimethylsulfoxide was added to dissolve the formazan crystals precipitates. After shaking the cell plate for 15 min, the absorbance(A) at a wavelength of 540 nm with a reference wavelength of 650 nm was measured with a Bio-Rad microplate reader. The relative cell viability (%) was calculated by $(A_{4T}/A_{blank}) \times 100$ (X values). The concentrations of 4T were calculated in log scales (Y values). Mean value and standard deviation were calculated using Graph Pad Prism version 7.0 (Graph Pad Software). Dose-response data table was chosen, with nonlinear regression the curve was gotten above. Error bars represent standard deviation. All samples were done in triplicate and the experiment was replicated three times.

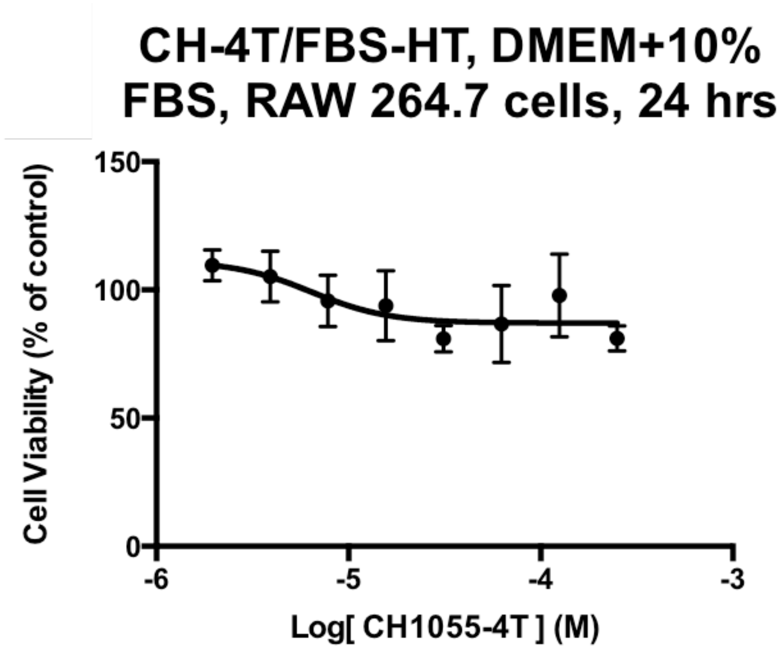

Supplementary Figure 19: Cell toxicity of CH-4T/FBS-HT. Evaluated using an MTT (3-(4,5- dimethylthiazol-2-yl)-2,5-diphenyl tetrazolium bromide) (Sigma-Aldrich, St. Louis, MO) assay. **Murine macrophage RAW 264.7 cells** plated into a 96 well plate at a concentration of $4*10^3$ cells/well in **DMEM with 10% fetal bovine serum (FBS)** at 37 ^oC and 5% CO^2, and 24 h later, the cells were incubated with CH-4T with different concentrations (250 μM, 125 μM, 62.5 μM, 31.25 μM, 15.63 μM, 7.81 μM, 3.91 μM, 1.95 μM, 0.98 μM) for 24 h. Old medium was taken out and new medium was added in 48 h later. Then 10 μL of the 12mM MTT stock solution was added and incubated for 3.5 hours. After removing medium, 150 μL dimethylsulfoxide was added to dissolve the formazan crystals precipitates. After shaking the cell plate for 15 min, the absorbance(A) at a wavelength of 540 nm with a reference wavelength of 650 nm was measured with a Bio-Rad microplate reader. The relative cell viability (%) was calculated by $(A_{4T}/A_{blank}) \times 100$ (X values). The concentrations of 4T were calculated in log scales (Y values). Mean value and standard deviation were calculated using Graph Pad Prism version 7.0 (Graph Pad Software). Dose-response data table was chosen, with nonlinear regression the curve was gotten above. Error bars represent standard deviation. All samples were done in triplicate and the experiment was replicated three times.
